# Supplementary material for: Analysis of Epichloë festucae small secreted proteins in the interaction with Lolium perenne
Source: PLoS One. 2019 Feb 13;14(2):e0209463. doi: 10.1371/journal.pone.0209463 (PMC6374014; doi:10.1371/journal.pone.0209463)
Supplement: S1 File — (PDF) [file pone.0209463.s001.pdf]

Supporting information: S1 File

14 November, 2018

Introduction

The Data

The analyses presented here combined data from a number of earlier studies, as cited in the Methods secton of the main paper. Data from each of these studies has been combined in a single file in the `data` directory of this repository.

```
library(tidyverse)
library(ggplot2)
library(ggrepel)

all_genes <- read_csv("data/combined_data.csv")
dim(all_genes)
```

```
## [1] 8367   21
```

As this dataframe has 21 columns, we briefly describe each one here:

```
knitr::kable(read_csv("cols.csv"))
```

Column	Description of data
gene	gene ID
length	protein length (aa)
nr__cysteines	number of cysteines
sp__classificatoin	signalP classification (non-secreted, secreted, ssp)
ep__classificatoin	EffectorP classification (NA for proteins > 200 aa)
ep__prob	EffectorP probability
apo__classificatoin	ApoplastP classification (NA for non-secreted proteins)
apo__prob	ApoplastP probability
expr__C__RPMK	Gene expression in culture
expr__P__RPMK	Gene expression in planta
PvC__qval	Q-value for differential expression culture v planta
PvC__L2FD	log2 fold difference in expression planta v culture
Hep__L2FD	log2 fold differnce in expression HepA del v WT
dAT	distance to nearest AT-rich sequence
MITE__nearest	nearest MITE
MITE__d	distance to neartest MITE
name	name of gene (used in plots)
Eathon__2015	was gene identified as ssp in Eaton 2015?
analysed	Was gene analysed by deletion mutatnt
sspcolumn	is gene a probable ssp (used inplots)

The key column for our analysis is `sp_classification`, which identifies small (< 200bp) secreted proteins (labeled `ssp`), larger secreted proteins (labeled `secreted`) and proteins that do not have a classical secretion signal (labeled `non-secreted`). As expected, only a small proportion of all genes ssps:

```r
knitr::kable(t(table(all_genes$sp_classification)))
```

non_secreted	secreted	ssp
7690	536	141

We will often use this classification as a point of comparison, so we can create a version of our data grouped by this column for future use.

```r
by_sp <- group_by(all_genes, sp_classification)
```

Helper functions

The file `utils.R` has some helper functions that we will used to compare fitted regression models and plot some results. Here we use source to import these functions.

```r
source('utils.R')
```

The proteins themselves

Are effectors cysteine rich?

We expect small secreted proteins to be active within the plant apoplast, where disulfide bridges formed between cysteine residues are known to stabilise protein structures. We thus begin by testing whether putative effectors are more cysteine-rich than other proteins. Specifically, we fit a logistic regression in which secretion class (i.e. non-secreted v secreted v small-secreted) predicts the proportion of amino acid residues that are cysteines.

A model with secretion class is a substantially better fit than a null model (every time we show these tales, the model with the lower AIC is the best-fitting one):

```r
null_cys <- glm(nr_cysteines/length ~ 1, data=all_genes, weights= length, family=binomial)

mod_cys <- glm(nr_cysteines/length ~ sp_classification,
               data=all_genes,
               weights= length,
               family=binomial)
AIC_table(mod_cys, null_cys)
```

	df	AIC	delta_AIC
mod_cys	3	51420.24	0.000
null_cys	1	52129.16	708.921

In addition, the secreted and small secreted proteins have a significantly higher proportion of cysteines than do other proteins. (here “estimate is an odds ratio).

```r
knitr::kable(tidy_glm(mod_cys, OR=TRUE))
```

term	estimate	std.error	statistic	p.value	conf.low	conf.high
secreted	1.275831	0.0165754	14.69637	6.801e-49	1.234870	1.317772

term	estimate	std.error	statistic	p.value	conf.low	conf.high
ssp	2.902185	0.0399875	26.64493	2.049e-156	2.680891	3.135921

Genome structure

Are MITES associated with secretion status?

A class of small DNA transposons called MITEs are associated with important biological functions in many fungal species. We have used the Fl1 reference genome to identify the nearest MITE to each of our protein coding genes. We begin by visualising this data:

```
plot_sp_classes(all_genes, MITE_d ) +
    scale_y_log10(label = function(x) x/1e3, "Distance from MITE (kbp)")
```

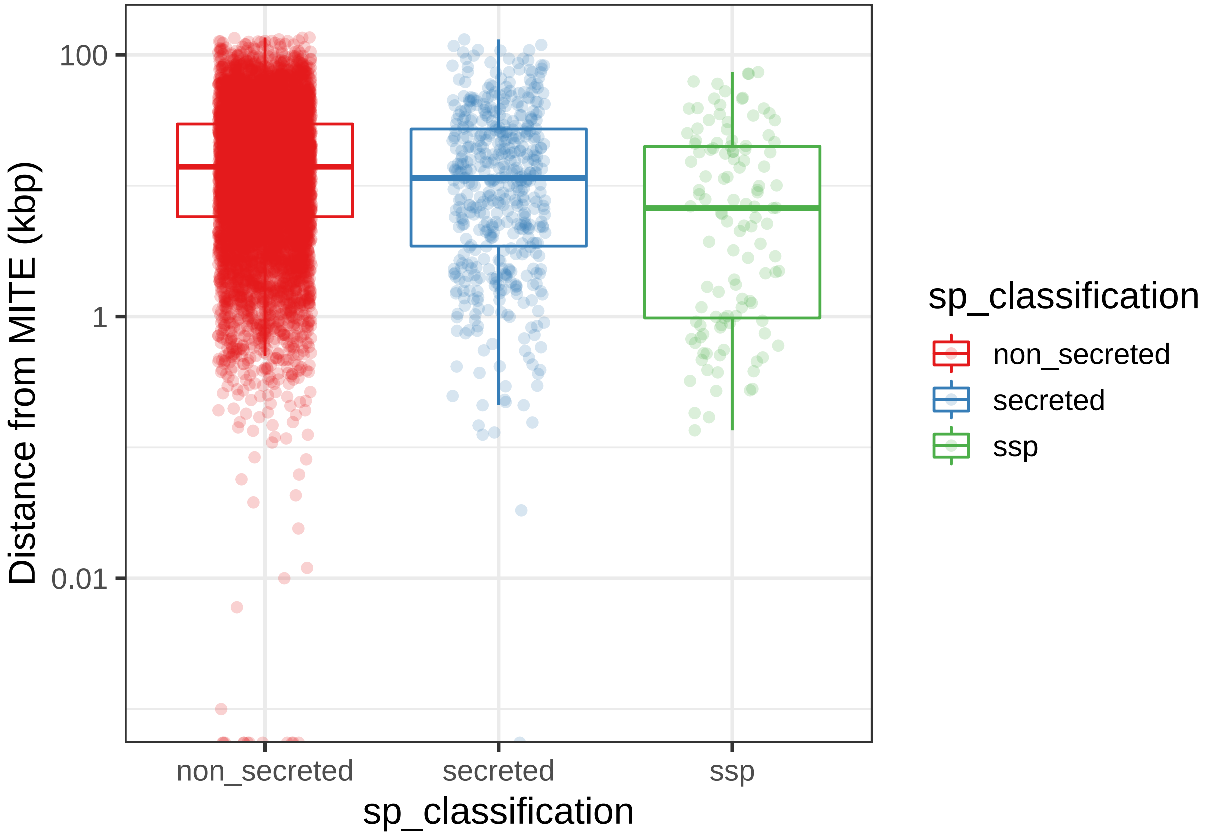

Clearly, the median distance between proteins and MITEs is lowest for ssps. If MITEs alter gene expression we expect them to act in *cis*. So we will focus in particular on MITEs near to the promotor region of a gene (that is 2kbp upsteram of a transcription start site) First we compare the proportion of genes with a MITE this close between secretion-classes:

```
mites <- summarise(by_sp, MITE_nearby= mean( MITE_d <= 2000, na.rm=TRUE))
knitr::kable(mites)
```

sp_classification	MITE_nearby
non_secreted	0.0917686

sp_classification	MITE_nearby
secreted	0.1656315
ssp	0.3589744

So, large secreted proteins are more likely to have a nearby MITE than non-secreted proteins and ssps are more than twice as likely again. We can put this to a more formal test by fitting a generalised linear model (in this case equivalent to logistic regression). First we can compare a model that using secretion classification as a predictor to one that does not:

```
mod_mite <-glm(MITE_d <= 2000 ~  sp_classification, data=all_genes, family=binomial)
null_mite <-glm(MITE_d <= 2000 ~  1, data=all_genes, family=binomial)
AIC_table(mod_mite, null_mite)
```

	df	AIC	delta_AIC
mod_mite	3	5002.680	0.00000
null_mite	1	5080.386	77.70632

Adding information of secretion classification substantially improves the fit of the model. We can use the `mod_mite` to measure the size and significance of this effect.

```
knitr::kable(tidy_glm(mod_mite, OR=TRUE))
```

term	estimate	std.error	statistic	p.value	conf.low	conf.high
secreted	1.964659	0.1290335	5.233671	1.662e-07	1.516484	2.516423
ssp	5.542303	0.1970053	8.692202	3.555e-18	3.738828	8.110933

The Fl1 genome contains a large number of MITE families. We can also test whether some MITE families are over-represented near to effectors using the chi squared test:

```
with_mite <-filter(all_genes, MITE_d < 2000)
chisq.test(with_mite$sp_classification, with_mite$MITE_nearest,
           simulate.p.value=TRUE, B=1e4
)
```

```
##
##  Pearson's Chi-squared test with simulated p-value (based on 10000
##  replicates)
##
## data:  with_mite$sp_classification and with_mite$MITE_nearest
## X-squared = 28.447, df = NA, p-value = 0.4329
```

A non-significant result means we cannot say particular MITE families are more or less likely to be near to putative effectors or other secreted proteins).

Are AT-rich or subtelomeric regions associated with ssps?

Epichloe genomes contain long contiguous chunks of AT-rich sequences. These sequences are associated with condensed chromatin state, and genes that are differentially expressed *in planta* are enriched near the edges of these regions. We can begin investigating a relationship between AT-rich regions and ssps by visualizing the distance between each gene and its nearest AT-rich region:

```r
ggplot(all_genes, aes(sp_classification, dAT, colour=sp_classification)) +
    geom_boxplot() +
    geom_jitter(height = 0, width = 0.2, alpha = 0.2)  +
    scale_color_brewer(palette = "Set1") + theme_bw() +
    scale_y_log10(label = function(x) x/1e3, "Distance from MITE (kbp)") +
    geom_label_repel(aes(label=analysed))
```

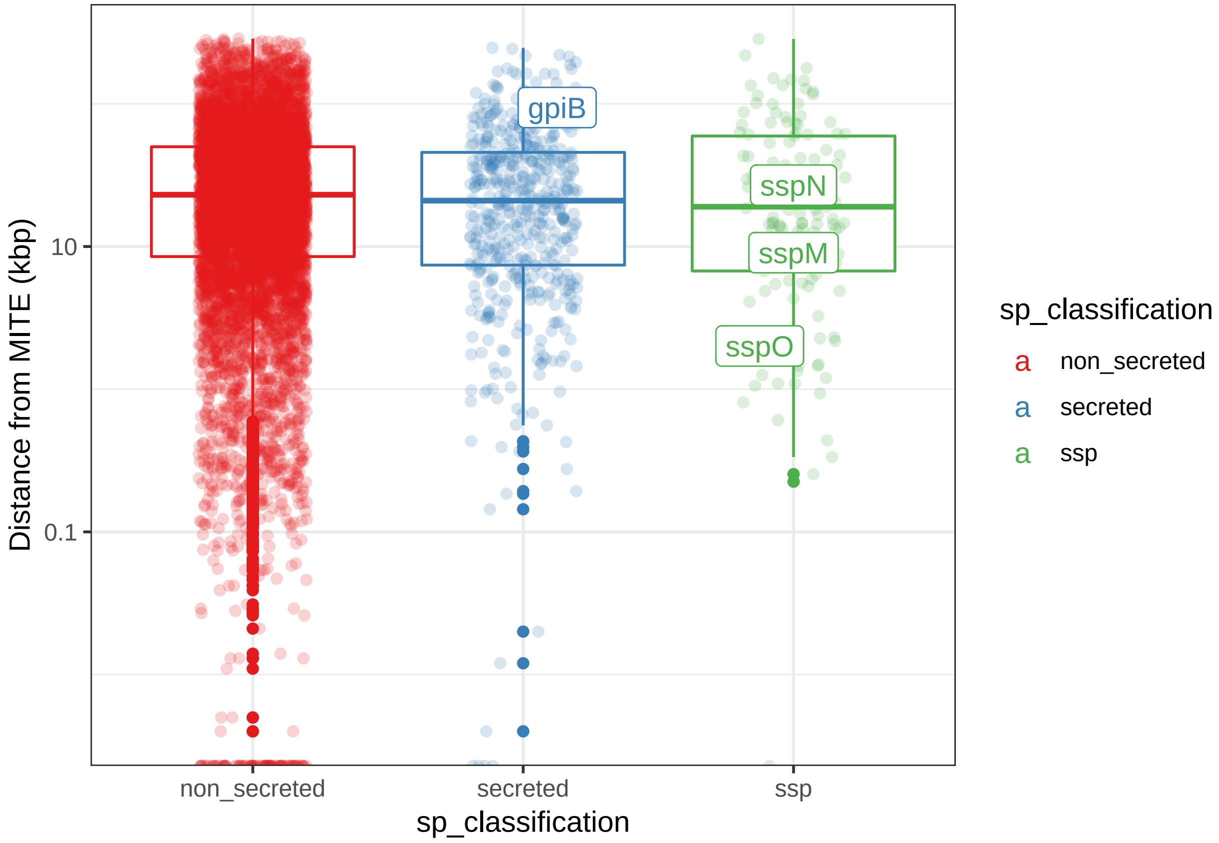

There are no obvious differences between secretion-classes, a result which is born out by more formal statistical testing.

```r
AT_mod <-glm(dAT <= 5000 ~  sp_classification, data=all_genes, family=binomial)

knitr::kable(tidy_glm(AT_mod))
```

term	estimate	std.error	statistic	p.value	conf.low	conf.high
secreted	0.0283918	0.1190976	0.2383911	0.8116	-0.2103653	0.2570172
ssp	0.1997843	0.2222504	0.8989154	0.3687	-0.2556238	0.6192676

A very similar pattern applies to telomeres (we use a 50kb cut-off here, as subtelomeric effects on expression and evolution of genes are generally on large scales such as this).

```r
telo_mod <-glm(dTelo <= 5e4 ~  sp_classification, data=all_genes, family=binomial)

knitr::kable(tidy_glm(telo_mod))
```

term	estimate	std.error	statistic	p.value	conf.low	conf.high
secreted	-0.2977436	0.4608846	-0.6460263	0.5183	-1.343024	0.5024005
ssp	0.1941951	0.7200768	0.2696867	0.7874	-1.615509	1.3592404

Do SSPs form clusters?

In some fungi, small secreted proteins appear in clusters of presumably co-regulated genes. Given that we have the location of all protein-coding genes in the Fl1 genome, we can test whether the small secreted ones are more likely to occur near to each other than we would expect by chance.

We start by identifying the locations of small secreted proteins using the file protein_data_locations. As the name suggests, this file has some summary data on each protein, including `prot_type` which assigns proteins to non-secreted, larged-secreted and small-secreted groups.

```
prot_data <- read_csv("data/protein_data_locations.csv")
prot_data
```

```
## # A tibble: 7,673 x 7
##    gene        prot_len secreted prot_type    chrom   start     end
##    <chr>          <int> <lgl>    <chr>        <chr>   <int>   <int>
##  1 EfM3.000020       36 FALSE    non-secreted chr1  6192755 6193707
##  2 EfM3.000030       43 FALSE    non-secreted chr1  6191513 6192773
##  3 EfM3.000040      391 FALSE    non-secreted chr1  6185437 6187551
##  4 EfM3.000050      372 FALSE    non-secreted chr1  6178846 6180714
##  5 EfM3.000060      191 FALSE    non-secreted chr1  6176666 6178198
##  6 EfM3.000070      344 FALSE    non-secreted chr1  6173968 6176299
##  7 EfM3.000080      168 FALSE    non-secreted chr1  6172663 6173964
##  8 EfM3.000090      126 FALSE    non-secreted chr3  5193150 5194880
##  9 EfM3.000110      203 FALSE    non-secreted chr3  5191499 5192908
## 10 EfM3.000120      284 FALSE    non-secreted chr3  5189243 5190517
## # ... with 7,663 more rows
```

Nearest-neighbour distance for small-secreted proteins

We want to know if the small-secreted proteins tend to come in 'clumps', or if they are even spread out along the genome. To start investigating this, we need to calculate the minimum distance to another SSP for each of our SSPs (i.e. the minimum within-group distance).

Thankfully the location data (the last three columns of the protein data) is in bed format, so we can used `bedtools` to calculate these distances. First, we need to write out a `bed` file for only the secreted proteins:

```
sort_bed <- function(x){
    x[order(x$chrom, x$start, x$end),]
}

secreted_loci <- select(
           filter(prot_data, prot_type=="small-secreted"),
           chrom, start, end, gene
)

write.simple.table(sort_bed(secreted_loci), "secreted_locs.bed")
```

Now we can use bedtools `closest` to get the nearest neighbour for each protein. Setting the `-io` flag means we ignore exact matches ('overlaps') so all distances are to another ssp. We also use `cut` to remove some

extraneous columns from the output.

```
bedtools closest -d -io -a secreted_locs.bed -b secreted_locs.bed  | \
  cut -f4,8,9 > secreted_neighbours.tsv
```

Now we can visualise the distribution of these within-group distances.

```
neigbours <- read_delim("secreted_neighbours.tsv", col_names=c("from", "to", "d"), delim="\t")
hist(neigbours$d, breaks="FD")
```

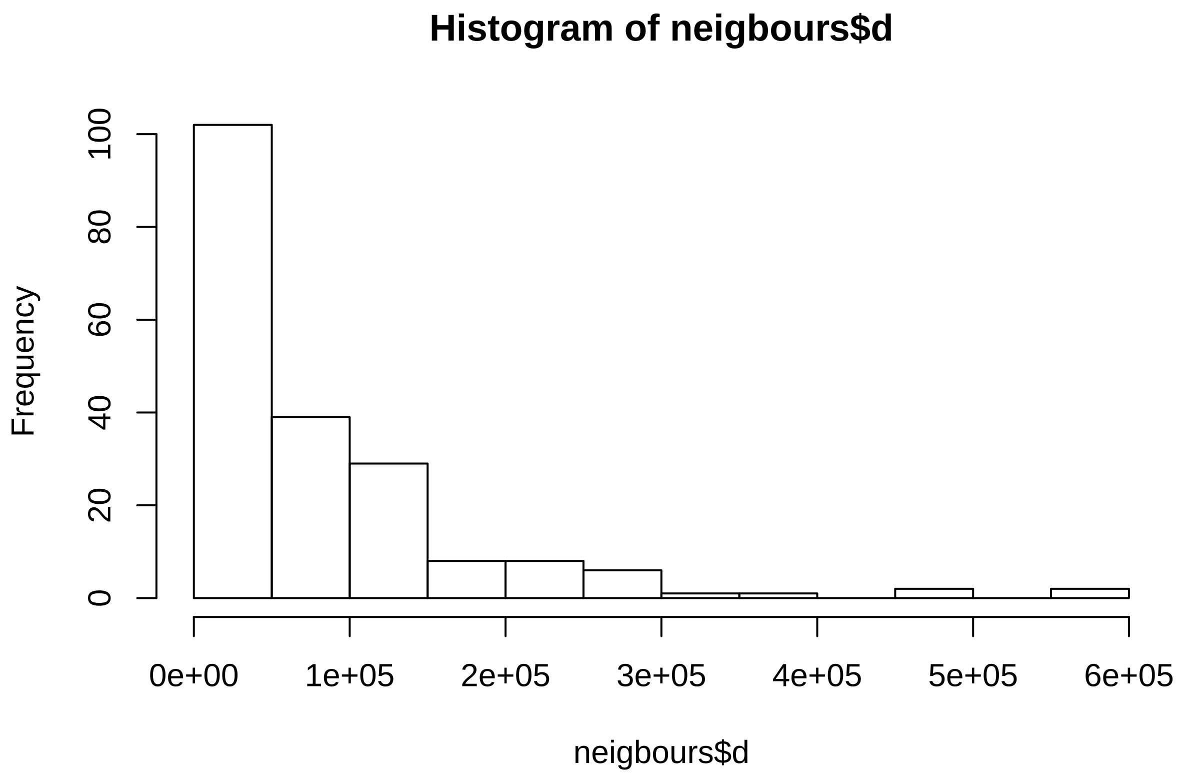

There are a few gene-pairs that fall close together (the left-most bin), but it certainly doesn't look like the ssps are very clustered. There are only four gene-pairs that fall within 1kb of other (note each pairing appears twice in this list):

```
knitr::kable(filter(neigbours, d < 1000))
```

from	to	d
EfM3.045120	EfM3.045110	940
EfM3.045110	EfM3.045120	940
EfM3.041760	EfM3.041770	793
EfM3.041770	EfM3.041760	793
EfM3.043290	EfM3.044640	640
EfM3.044640	EfM3.043290	640
EfM3.008740	EfM3.008750	655
EfM3.008750	EfM3.008740	655

Looking at these in the genome , two pairs (EfM3.045110-EfM3.045120 and EfM3.043290-EfM3.044640) are

divergently transcribed. The others two are transcribed from the same strand.

Simulating a null distribution

To know if these genes are more clustered than we might expect by chance we need to simulate a null distribution. We do that by repeating the procedure we used to calculated within-group neighbour distances for random subsets of genes, each the same size as SSP dataset.

Here I select all the non SSP proteins, and write the locations of 1 000 random subsets of these genes…

```
write_random_subset <- function(full_data, n, replicate){
    fname <- paste0("null_distr/rep_", i, ".tsv")
    write.simple.table( sort_bed(sample_n(full_data, n)), fname)
    return(TRUE)
}

other_loci <- select(filter(prot_data, prot_type!="small-secreted"), chrom, start, end, gene)

dir.create("null_distr")
for( i in 1:1000 ){
   write_random_subset(other_loci, 220, i)
}
```

Then, using `bedtools` and `bash` calculate the distance distributions. Note I am only keeping the distance column in this case:

```
#iterative over all of the samples written above
for f in null_distr/*.tsv
do
    # for each one, calculate the nearst sub-sampled gene and write the distance
    # column of the output to a new file
    bedtools closest -d -io -a $f -b $f | cut -f9 > ${f%.*}_distances.list
done
```

We can now compare our observed data to the null distribution. First we can consider the mean distance between SSP in our data compare to the null distribution.

```
null_files <- list.files("null_distr/", pattern="distances.list", full.names=TRUE)
null_d <- lapply(null_files, scan, quiet=TRUE)
hist(sapply(null_d, mean),
     main="Null distribution of mean nearest-neighbour distance",
     xlab="Mean distance to nearest gene")
abline(v=mean(neigbours$d), col="red", lwd=3)
```

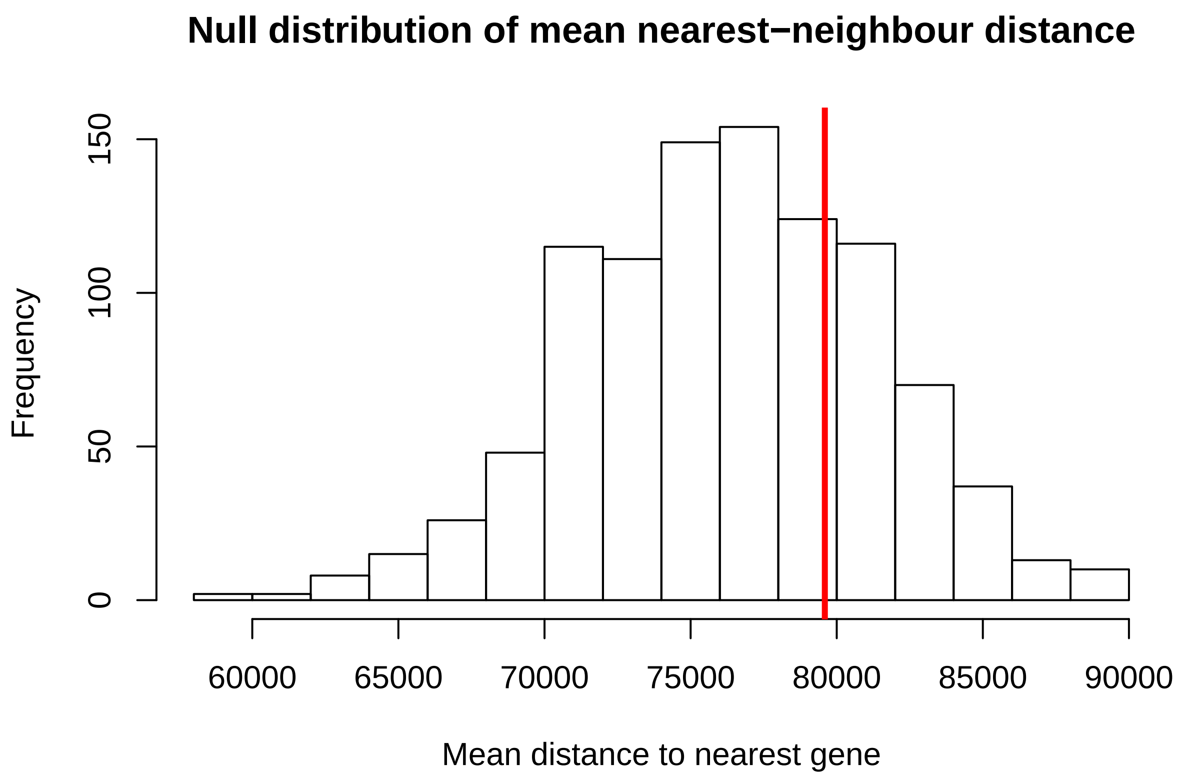

In this graph the histogram shows the null distribution and the read line our observed data. So, we find no evidence that SSP are on average closer to each other than we'd exect by chance. Here is a p-value for the test.

```
mean(mean(neigbours$d) < sapply(null_d, mean))
```

```
## [1] 0.272
```

Gene expression

Are effectors differentially expressed *in planta*

Effectors are often lowly-expressed or silenced when fungi are grown in axenic culture, and only highly expressed when they infect a plant. We can used previously published RNAseq data to investigate whether putative effectors are more highly expressed *in planta*

We use the log2 fold difference in gene expression as summary statistic for each gene (here positive numbers represent higher expression *in planta*).

```
PvC_summ <- summarise(by_sp,
              mean_L2DF = mean(PvC_L2FD, na.rm=TRUE),
              prop_gt_2 = mean(PvC_L2FD > 2 & PvC_qval < 0.01, na.rm=TRUE),
              n_gt_2    = sum(PvC_L2FD > 2 & PvC_qval < 0.01, na.rm=TRUE)
)
knitr::kable(PvC_summ)
```

sp_classification	mean_L2DF	prop_gt_2	n_gt_2
non_secreted	-0.0044065	0.0652174	477
secreted	0.2005926	0.1232604	62
ssp	1.1965866	0.3467742	43

So, indeed, putative effectors have over-all higher gene expression *in planta* (mean > 1 unit of log2 fold difference) and higher proportion of these genes have substantial (> 2) and significant differences in expression.

Are effectors released from repression in HepA deletion strains?

In many species, chromatin-state contributes to the regulation of effectors. We can use RNAseq data from a Heterochromatin protein 1 (*hepA*) knockout strain to investigate whether the absence of this protein releases effector from expression in culture.

At first glance, there appears to be little difference among gene classes:

```
plot_sp_classes(all_genes, Hep_L2FD) +
    ylab("L2FD in hepA deletion mutant")
```

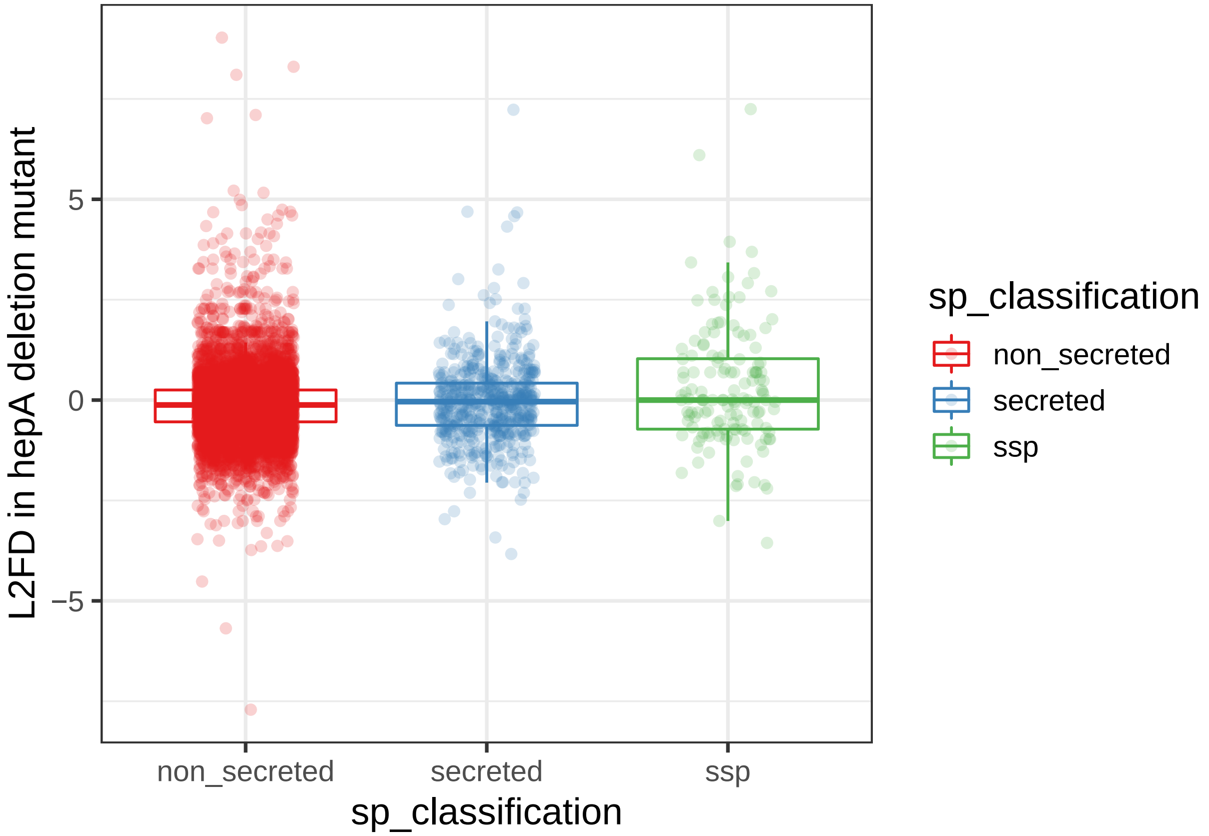

However, a considerably higher proportion of effector genes have a substantial increase in gene expression in culture in the *hepA* deletion strain

```
hepA_summ <- summarise(by_sp,
              mean_L2DF = mean(Hep_L2FD, na.rm=TRUE),
              prop_gt_2 = mean(Hep_L2FD > 2, na.rm=TRUE),
              n_gt_2    = sum(Hep_L2FD > 2, na.rm=TRUE)
```

```
)
knitr::kable(hepA_summ)
```

sp_classification	mean_L2DF	prop_gt_2	n_gt_2
non_secreted	-0.1177579	0.0152821	117
secreted	-0.0409798	0.0299625	16
ssp	0.3099269	0.1142857	16

And a statistical test confirms this difference is highly significant.

```
mod_hepA <- glm(Hep_L2FD > 2 ~ sp_classification, data=all_genes, family=binomial)
knitr::kable(tidy_glm(mod_hepA))
```

term	estimate	std.error	statistic	p.value	conf.low	conf.high
secreted	0.6882844	0.2703889	2.545535	0.01091	0.1209911	1.187980
ssp	2.1179780	0.2815034	7.523810	5.32e-14	1.5319714	2.642563

How is effector expression altered in symbiosis mutants?

Ee can recreate figure 2C from the paper, comparing the expression of different gene classes across four different symbiosis mutants *in planta*.

```
mutant_expr <- read_csv("data/mutant_expr_summary.csv")

ggplot(mutant_expr, aes(mutant , Log2_fold_diff , colour=sp_classification, label=analysed)) +
    geom_boxplot(outlier.shape=NA) +
    scale_colour_brewer(palette="Set1") +
    geom_point(position=position_jitterdodge(jitter.width=0.1, jitter.height=0), alpha=0.4) +
    xlab("Mutant") +
    scale_y_continuous("Log2 Fold Difference in expressoin", limits=c(-12,12)) +
    geom_label(position=position_dodge(1))
```

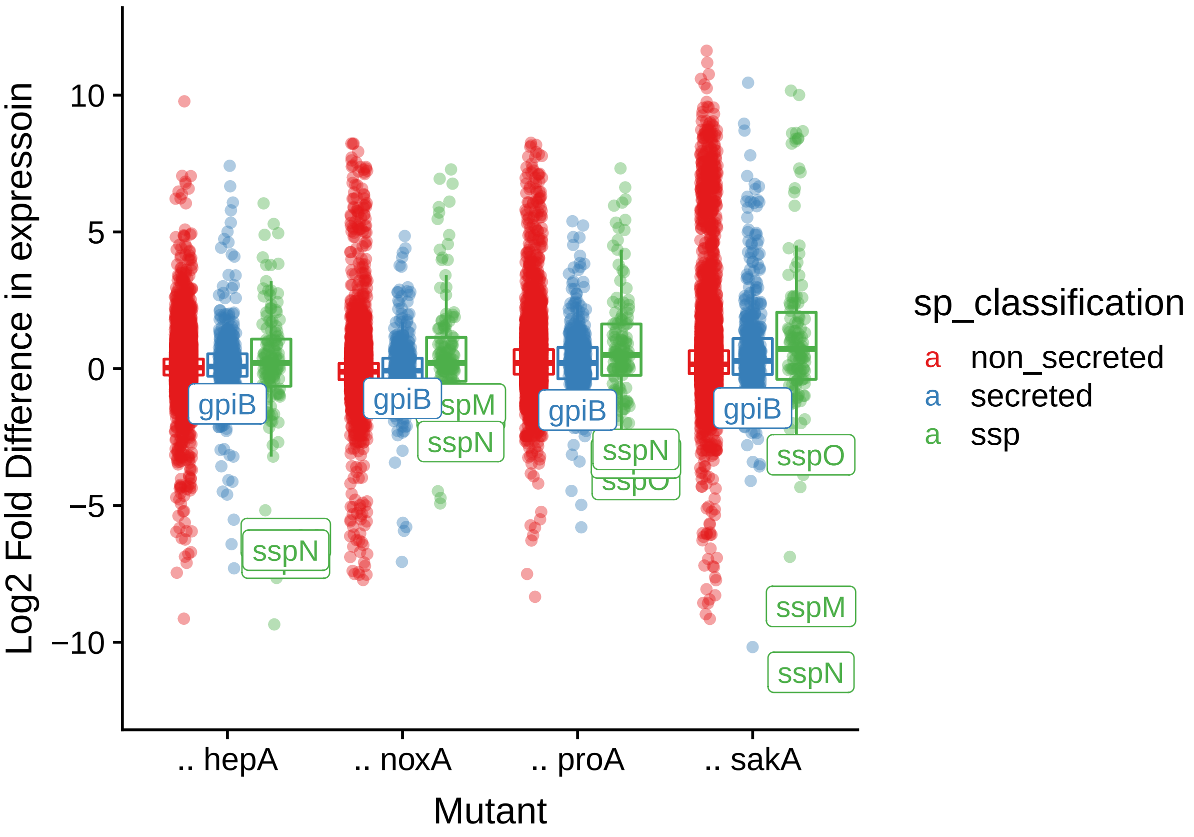

Plant phenotype analyses.

Finally, we will analyse the plant phenotype data discussed in the man text of the paper. In each case we will compare grasses infected with genetically modified fungi (either deletion or over-expression strains) to those infected by wild-type fungi (here labeled FL1).

Tiller number

Starting with data on the number of tillers in a plant. Visually, there is little evidence for a difference between the wild type and any of the mutants.

```
library(cowplot)
OE_tiller_num <- read_plant_data("data/OE_tiller_num.csv")
DEL_tiller_num <- read_plant_data("data/del_tiller_num.csv")

num_A <- plant_plot(OE_tiller_num, "") +
    scale_y_continuous("N. tillers",limits=c(0,20))
num_B <- plant_plot(DEL_tiller_num, "") +
    scale_y_continuous(limits=c(0,20))
plot_grid(num_A, num_B, labels=c("oe", "del"))
```

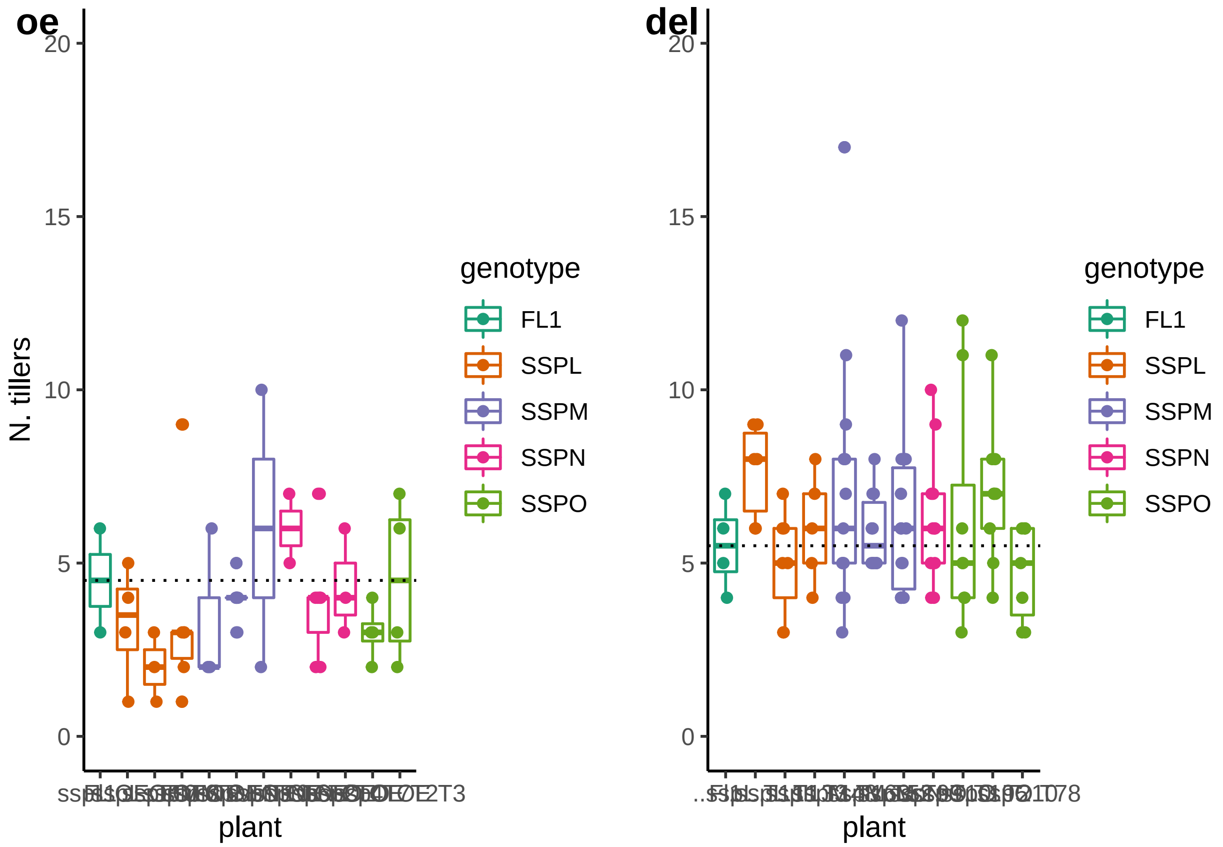

Performing a one-way ANOVA, we find no evidence for difference among genotypes in the over-expression strains…

```
mod_OE_n <- lm(measure ~ genotype, data=OE_tiller_num)
anova(mod_OE_n)
```

```
## Analysis of Variance Table
##
## Response: measure
##           Df  Sum Sq Mean Sq F value Pr(>F)
## genotype   4  12.709  3.1772   0.779 0.5453
## Residuals 41 167.226  4.0787
```

… and the estimated effect sizes are not statistically significant.

```
knitr::kable(tidy_glm(mod_OE_n))
```

term	estimate	std.error	statistic	p.value	conf.low	conf.high
genotypeSSPL	-1.4230769	1.533978	-0.9277037	0.359	-4.521008	1.674854
genotypeSSPM	-0.3181818	1.552460	-0.2049533	0.8386	-3.453439	2.817075
genotypeSSPN	-0.1666667	1.542476	-0.1080514	0.9145	-3.281761	2.948428
genotypeSSPO	-0.7500000	1.596615	-0.4697439	0.641	-3.974429	2.474429

Results for the deletion strain are similar to the over-expression strain.

```
mod_DEL_n <- lm(measure ~ genotype, data=DEL_tiller_num)
anova(mod_DEL_n)
```

```
## Analysis of Variance Table
##
## Response: measure
##           Df Sum Sq Mean Sq F value Pr(>F)
## genotype   4   4.25  1.0637  0.1906 0.9427
## Residuals 89 496.60  5.5797
```

Although the estimate for each strain is positive, none of them are statistically significant.

```
knitr::kable(tidy_glm(mod_OE_del))
```

term	estimate	std.error	statistic	p.value	conf.low	conf.high
genotypeSSPL	0.6666667	1.305725	0.5105719	0.6109	-1.927782	3.261115
genotypeSSPM	0.9324324	1.243277	0.7499798	0.4552	-1.537932	3.402797
genotypeSSPN	0.7727273	1.379196	0.5602737	0.5767	-1.967706	3.513160
genotypeSSPO	0.5833333	1.275705	0.4572637	0.6486	-1.951464	3.118131

Tiller length

Next, we consider data on the length of tillers. Again, there is little evidence for differences in any strain.

```
OE_tiller_len <- read_plant_data("data/OE_tiller_len.csv")
DEL_tiller_len <- read_plant_data("data/del_tiller_len.csv")

len_A <- plant_plot(OE_tiller_len, "") +
    scale_y_continuous("tiller length",limits=c(0,600))
len_B <- plant_plot(DEL_tiller_len, "") +
    scale_y_continuous(limits=c(0,600))
plot_grid(len_A, len_B, labels=c("oe", "del"))
```

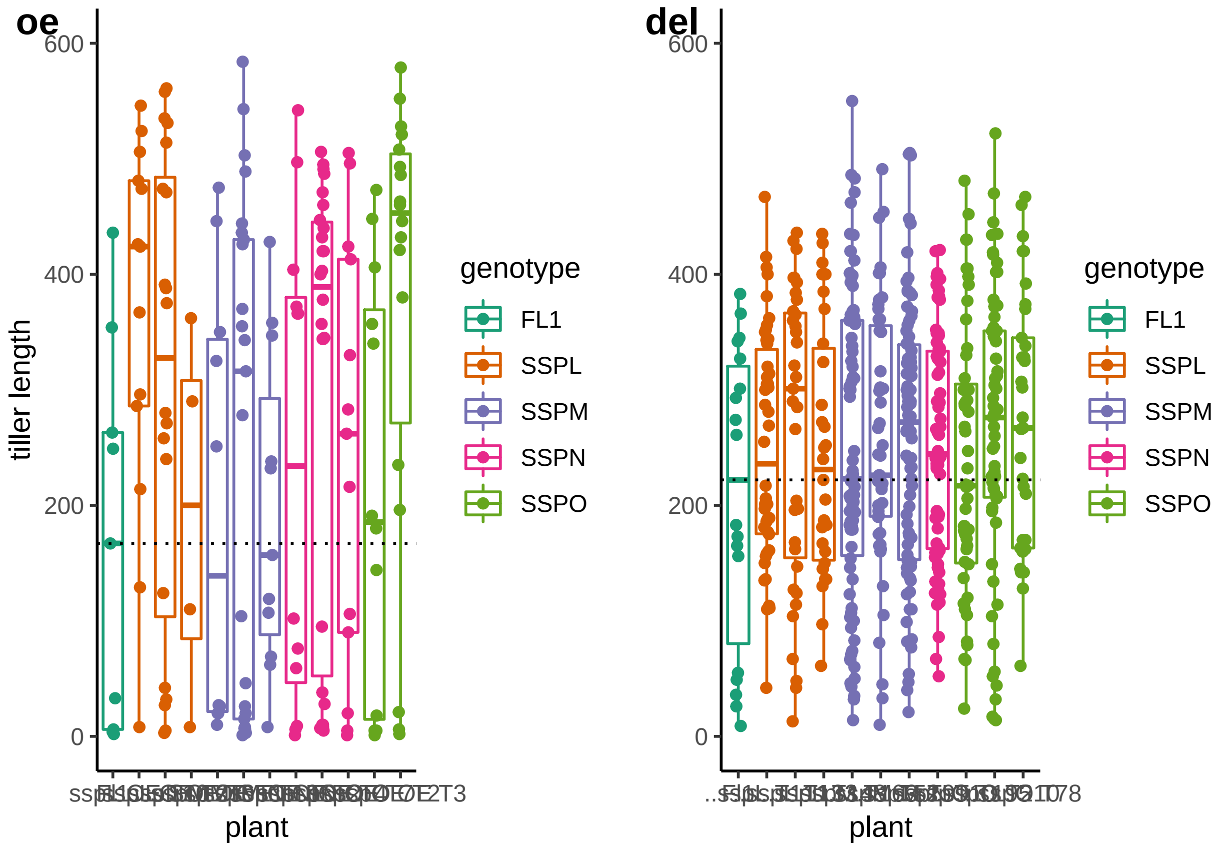

ANOVA for the over-expression strains.

```
mod_OE_len <- lm(measure ~ genotype, data=OE_tiller_len)
anova(mod_OE_len)
```

```
## Analysis of Variance Table
##
## Response: measure
##            Df  Sum Sq Mean Sq F value  Pr(>F)
## genotype    4  336366   84092  2.2128 0.06965 .
## Residuals 170 6460481   38003
## ---
## Signif. codes:  0 '***' 0.001 '**' 0.01 '*' 0.05 '.' 0.1 ' ' 1
```

The naive p-value for strain *sspL* is < 0.05.

```
knitr::kable(tidy_glm(mod_OE_len))
```

term	estimate	std.error	statistic	p.value	conf.low	conf.high
genotypeSSPL	151.06725	72.26764	2.0903860	0.03807	8.409731	293.7248
genotypeSSPM	54.88647	71.05410	0.7724603	0.4409	-85.375508	195.1485
genotypeSSPN	108.98932	70.38011	1.5485811	0.1233	-29.942204	247.9208
genotypeSSPO	141.67778	74.08980	1.9122442	0.05753	-4.576716	287.9323

However, we performed four independent tests (each genotype v WT). After adjusting for multiple testing, the effect is no-longer significant.

```r
mod_summ <- tidy_glm(mod_OE_len)
mod_summ$padj <- p.adjust(mod_summ$p.value, method="bonferroni")
knitr::kable(mod_summ)
```

term	estimate	std.error	statistic	p.value	conf.low	conf.high	padj
genotypeSSPL	151.06725	72.26764	2.0903860	0.03807	8.409731	293.7248	0.15228
genotypeSSPM	54.88647	71.05410	0.7724603	0.4409	-85.375508	195.1485	1.00000
genotypeSSPN	108.98932	70.38011	1.5485811	0.1233	-29.942204	247.9208	0.49320
genotypeSSPO	141.67778	74.08980	1.9122442	0.05753	-4.576716	287.9323	0.23012

Finally, we consider the deletion strains. The one-way ANOVA is no significantly different from a null model.

```r
mod_DEL_len <- lm(measure ~ genotype, data=DEL_tiller_len)
anova(mod_DEL_len)
```

```
## Analysis of Variance Table
##
## Response: measure
##            Df  Sum Sq Mean Sq F value Pr(>F)
## genotype    4   36767  9191.7  0.6876 0.6007
## Residuals 557 7445447 13367.1
```

And no effects are significant.

```r
knitr::kable(tidy_glm(mod_DEL_len))
```

term	estimate	std.error	statistic	p.value	conf.low	conf.high
genotypeSSPL	45.00000	29.37750	1.531784	0.1261	-12.704238	102.7042
genotypeSSPM	45.46330	28.35367	1.603436	0.1094	-10.229888	101.1565
genotypeSSPN	42.54412	30.64618	1.388236	0.1656	-17.652088	102.7403
genotypeSSPO	46.85714	28.87120	1.622972	0.1052	-9.852597	103.5669
