# Supplementary figures and images for: Analysis of Epichloë festucae small secreted proteins in the interaction with Lolium perenne
Source: PLoS One. 2019 Feb 13;14(2):e0209463. doi: 10.1371/journal.pone.0209463 (PMC6374014; doi:10.1371/journal.pone.0209463)

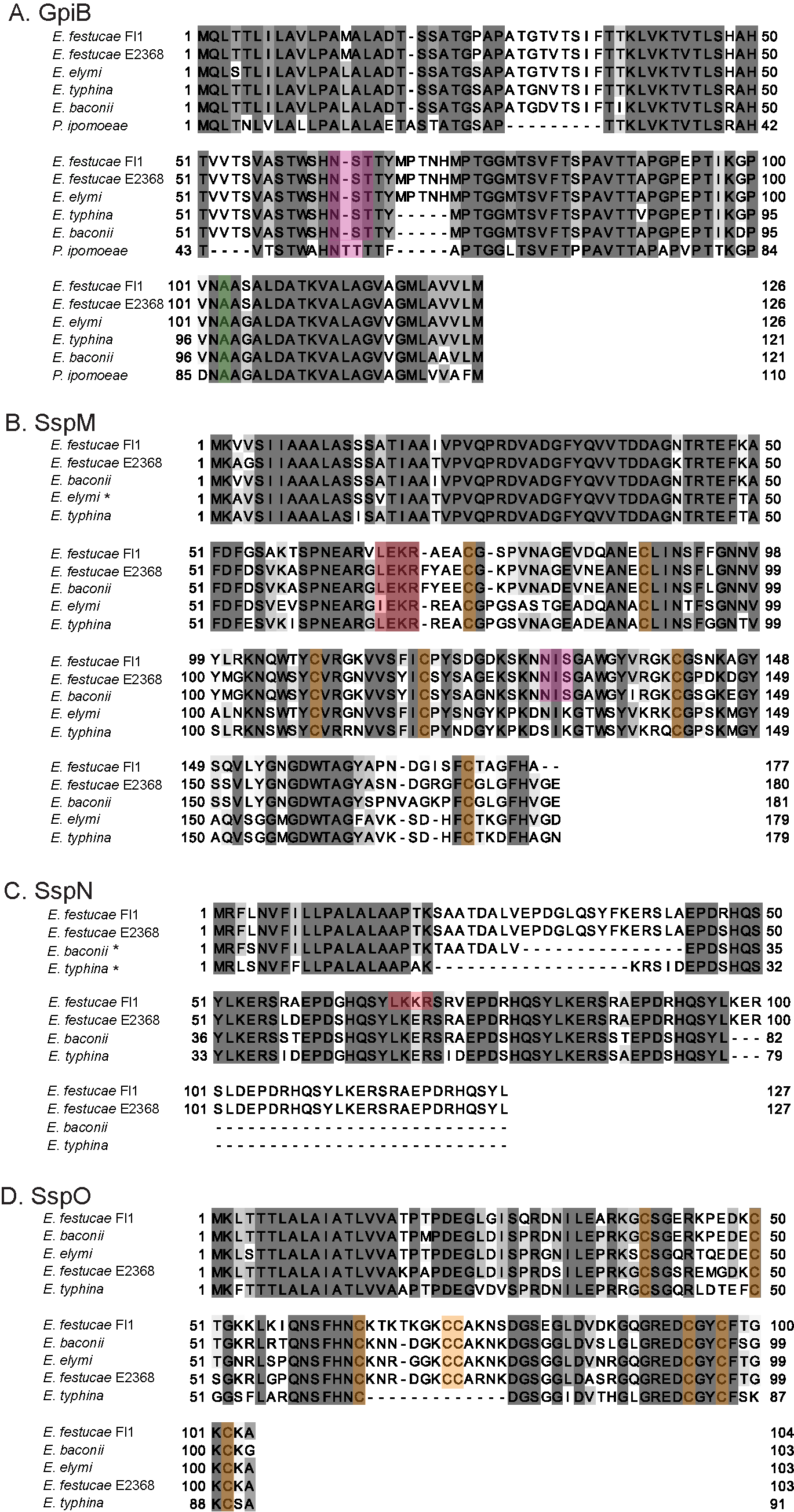

Supplement: S1 Fig — Alignments of GpiB (A), SspM (B), SspN (C) and SspO (D) with homologs from a selection of Epichloë species. Red shading: Protease cleavage site; Green shading: omega site; Purple shading: N-glycosylation site; Orange shading: cysteine residues. Protein sequences for E. baconii, E. elymi and E. typhina were obtained from the Kentucky Endophyte database when available or predicted using FGENESH using Claviceps purpurea parameters. The deduced amino acid sequence was used for the alignment. Sequences marked with * were manually annotated. (TIF) [file pone.0209463.s002.tif]

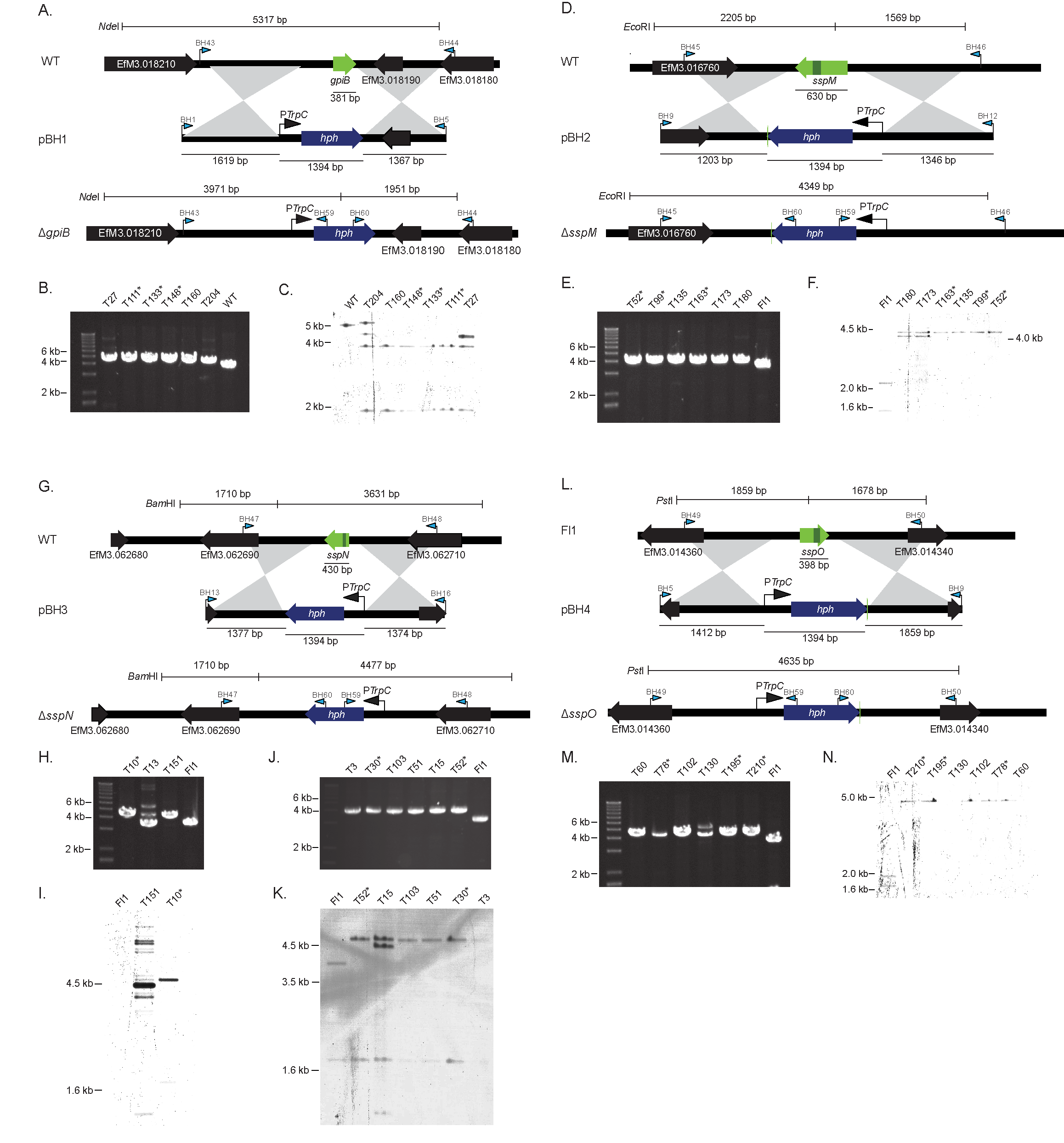

Supplement: S2 Fig — (A) Physical map of the gpiB WT genomic locus, linear insert of the gpiB replacement construct, pBH1, and the recombinant locus showing restriction enzyme sites for NdeI. Grey shading indicates regions of recombination. Numbers indicate the PCR primer pairs used for Gibson assembly (BH1/BH5) and deletion mutant screening (BH43/BH44). (B) PCR screening of deletion candidates with the PCR primer pair BH43/BH44, generated expected bands of 3,997 bp in WT, and 4,602 bp in deletion mutants (C) NBT/BCIP-stained Southern blot of digests (approx. 1 μg) from E. festucae WT, ΔgpiB T27, ΔgpiB T111 (PN3113), ΔgpiB T133 (PN3114), ΔgpiB T148 (PN3115), ΔgpiB T160 and ΔgpiB T204 strains probed with digoxigenin (DIG)-11-dUTP-labeled linear insert of pBH1 amplified with the primer pair BH1/BH5. Expected bands of 5,317 bp in WT, and 3,971 bp and 1,951 bp in the deletion mutant. (D) Physical map of the sspM WT genomic locus, linear insert of the sspM replacement construct, pBH2, and the recombinant locus showing restriction enzyme sites for EcoRI. Grey shading indicates regions of recombination. Numbers indicate the PCR primer pairs used for Gibson assembly (BH9/BH12) and deletion mutant screening (BH45/BH46). (E) PCR screening of deletion candidates with the PCR primer pair BH45/BH46, generated expected bands of 3,615 bp in WT, and 4,190 bp in deletion mutants. (F) NBT/BCIP-stained Southern blot of digests (approx. 1 μg) of E. festucae WT, ΔsspM T180, ΔsspM T173, ΔsspM T163 (PN3118), ΔsspM T135, ΔsspM T99 (PN3117) and ΔsspM T52 (PN3116) strains probed with digoxigenin (DIG)-11-dUTP-labeled linear insert of pBH2 amplified with the primer pair BH9/BH12. Expected bands of 2,205 bp and 1,569 bp in WT and 4,349 bp in the deletion mutant. (G) Physical map of the sspN WT genomic locus, linear insert of the sspN replacement construct, pBH3, and the recombinant locus showing restriction enzyme sites for BamHI. Grey shading indicates regions of recombination. Numbers indicate the PCR primer pai [file pone.0209463.s003.tif]

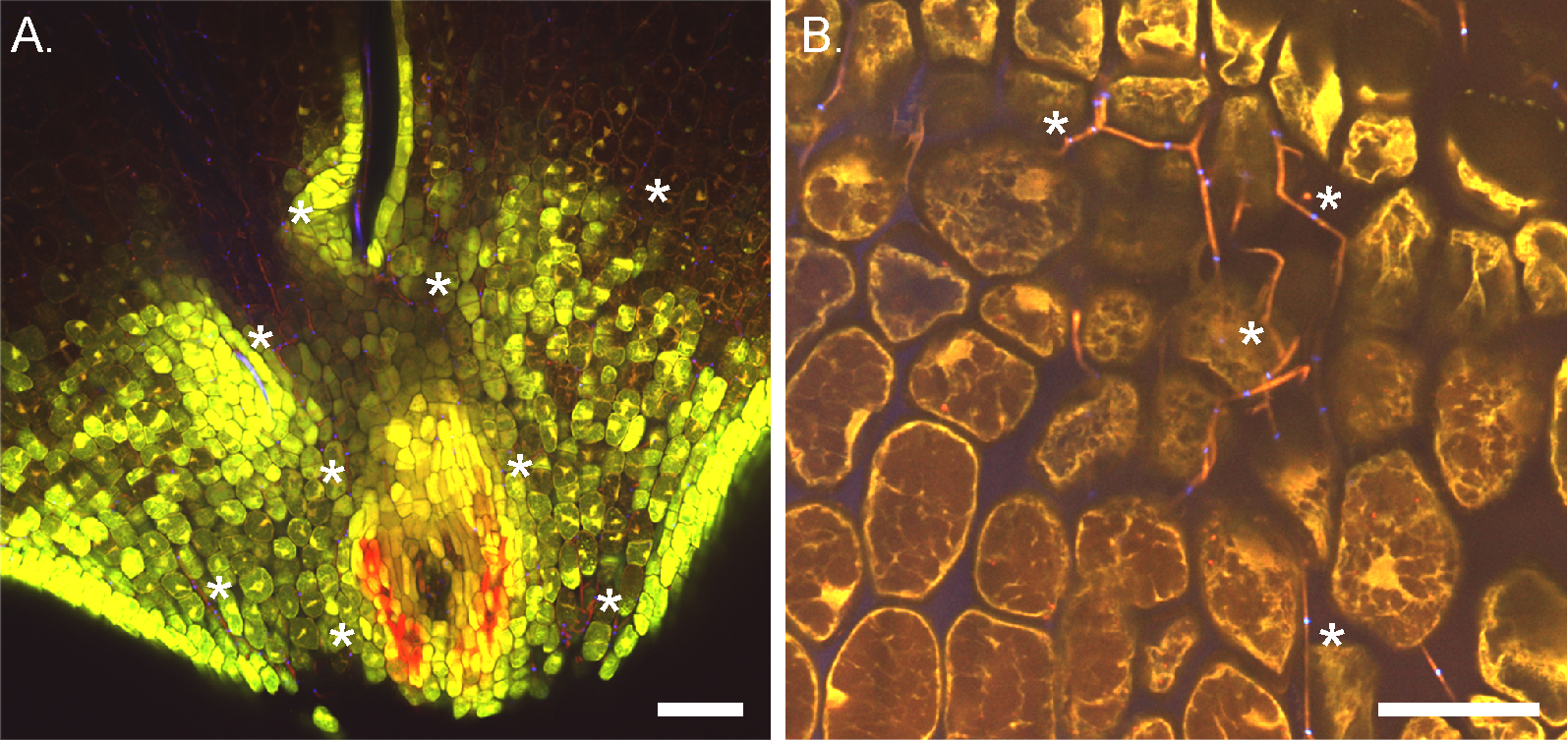

Supplement: S3 Fig — The fungal endophytic cell wall was stained with aniline blue (orange pseudo colour) while fungal septa were stained with WGA-AF 488 (blue pseudo colour). A. Ovary of mutant strain ΔsspM T52, B. Ovary of mutant strain ΔsspM T163. E. festucae hyphae are marked by asterisk. Scale bar: 50 μm. (TIF) [file pone.0209463.s004.tif]

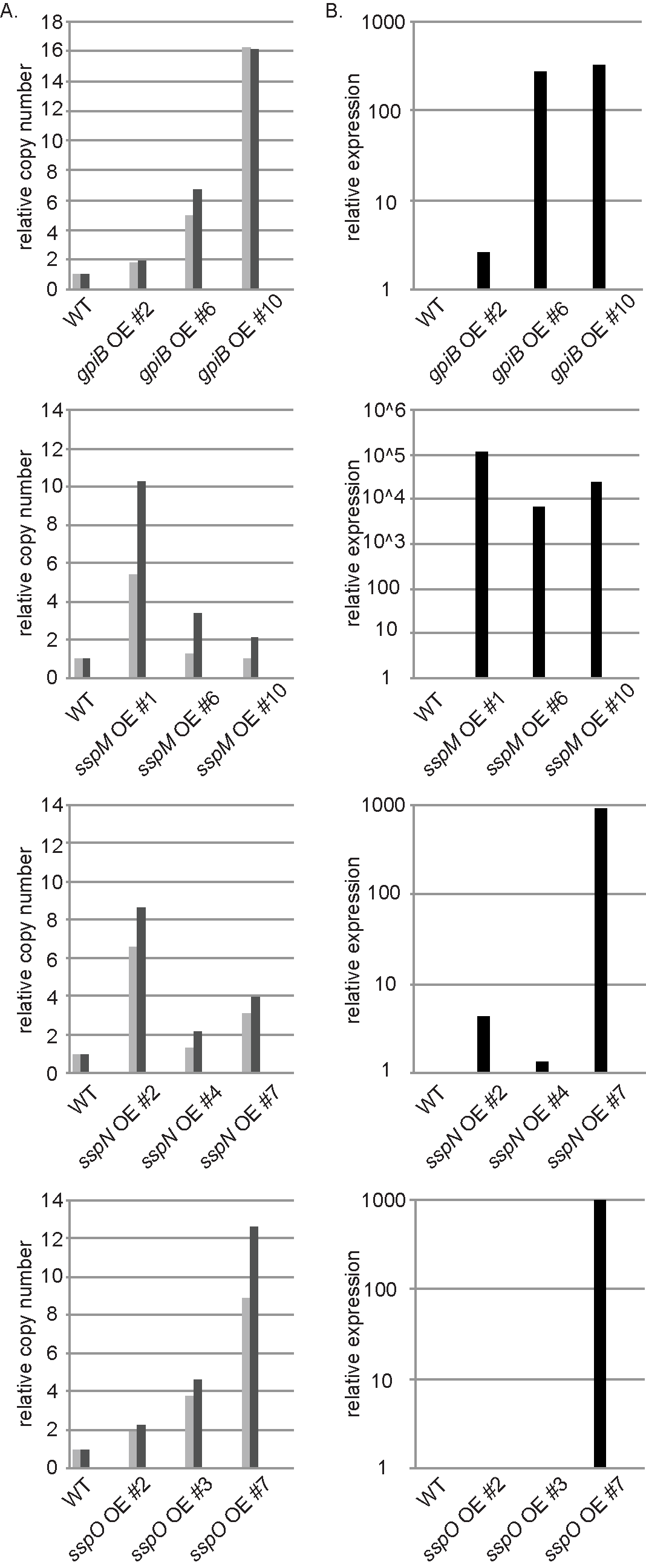

Supplement: S4 Fig — (A) Copy number determined by qPCR is expressed relative to the WT copy number. Genes encoding hepA (single copy, light grey) and pacC (single copy, dark grey) were used as reference genes. (B) Expression level determined by RT-qPCR is expressed relative to the WT gene expression. The 40S ribosomal S22 gene was used as reference gene. Primers used for the analyses are given in S2 Table. (TIF) [file pone.0209463.s005.tif]

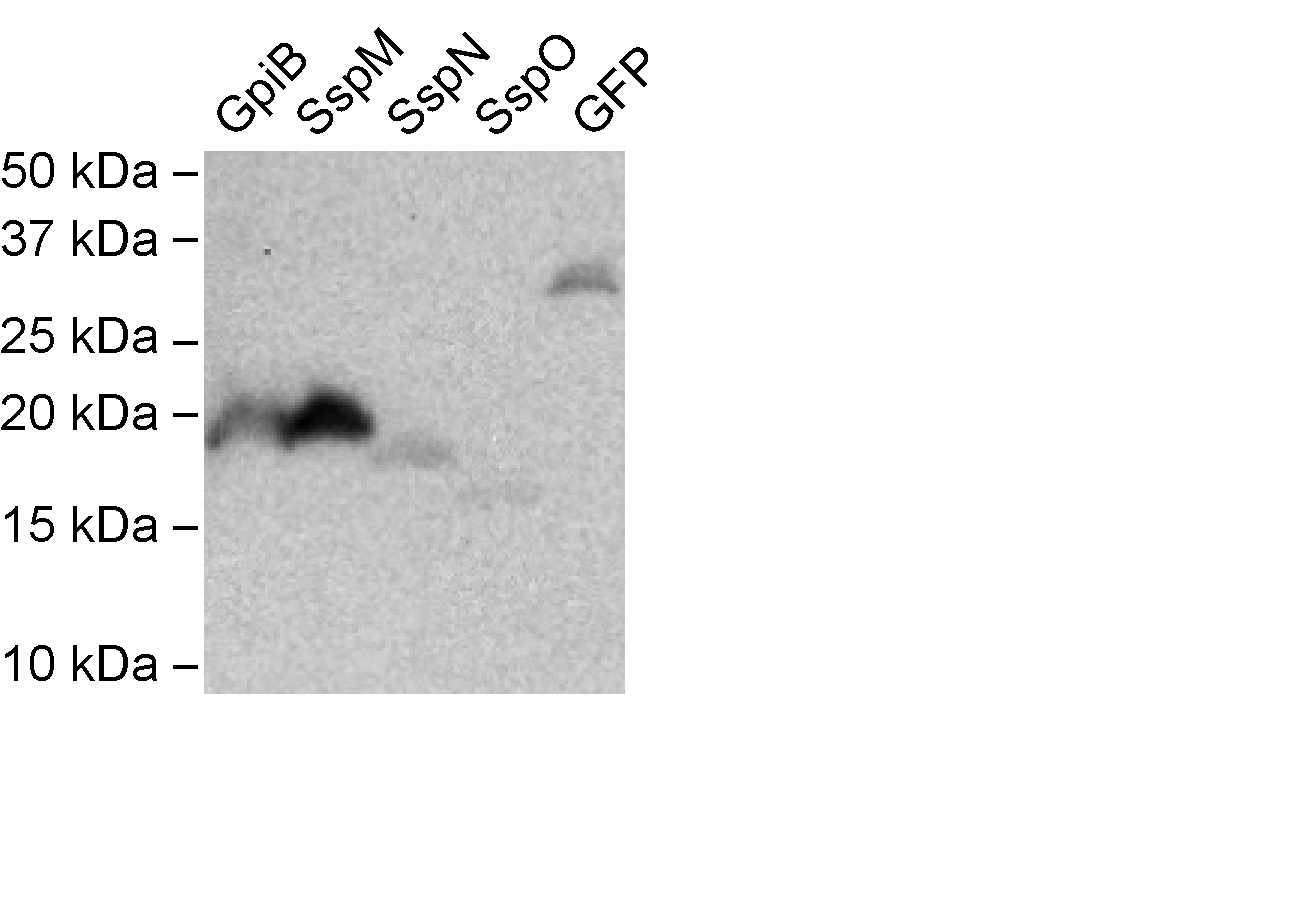

Supplement: S5 Fig — Total protein of the infiltrated leaf region was extracted and separated by electrophoresis on a 10% SDS gel. The gel was transferred to a membrane and probed with an anti-FLAG antibody. eGFP expressed in N. benthamiana served as positive control. Expected sizes: approx. 9.8 kDa for GpiB, 14 kDa for SspN, 10 kDa for SspO and for SspM 14.8 and 18.8 kDa. (TIF) [file pone.0209463.s006.tif]
