# Supplementary material for: Analysis of Epichloë festucae small secreted proteins in the interaction with Lolium perenne
Source: PLoS One. 2019 Feb 13;14(2):e0209463. doi: 10.1371/journal.pone.0209463 (PMC6374014; doi:10.1371/journal.pone.0209463)
Supplement: S2 Table — (DOCX) [file pone.0209463.s008.docx]

**Table S2.** Biological material.

| **Organism/Strain** | **Characteristics** | **Reference** |
| --- | --- | --- |
| ***E. coli*** | | |
| DH5α | F^–^, φ80*lacZ*, ΔM15, Δ(*lacZYA*-*argF*), U169, *recA1*, *endA1*, *hsdR17* (r_k_^–^, m_k_^–^), *phoA*, *supE44*, λ^–^, *thi-1*, *gyrA96*, *relA1* | Invitrogen |
|  |  |  |
| ***A. tumefaciens*** | | |
| GV3101 | C58 (Rif^R^), Ti pMP90 (pTiC58DT-DNA) (Gen^R^/Str^R^), Nopaline | Koncz et al. 1994 |
| RB53 | GV3101/pICH86988 | Melissa Guo |
| RB28 | GV3101/ pBC302-3-R3a | Segretin et al. 2014 |
| RB29 | GV3101/pBIN-Plus-Avr3a | This study |
| RB45 | GV3101/pBC302-3-INF1 | This study |
| RB46 | GV3101/pICH86966-N-3xFLAG-GFP (3xFLAG-GFP) | This study |
|  | GV3101/pBH44 (PRIα-*gpiB*) | This study |
|  | GV3101/pBH45 (PRIα-*sspM*) | This study |
|  | GV3101/pBH46 (PRIα-*sspN*) | This study |
|  | GV3101/pBH47 (PRIα-*sspO*) | This study |
|  |  |  |
| ***S. cerevisiae*** | | |
| PN2965 (YTK12) | *MATα* *ura3-52* l*eu2*Δ*1* *trp1*-Δ*63* *his3*-Δ*200* *lys2*-Δ*202* | [Winston et al. 1995](#_ENREF_3) |
|  | YTK12/pSUC2TM13ORI (SUC2 without SS) | This study |
|  | YTK12/pBH5 (SS-*gpiB*-SUC2) | This study |
|  | YTK12/pBH11 (SS-*sspM*-SUC2) | This study |
|  | YTK12/pBH9 (SS-*sspN*-SUC2) | This study |
|  | YTK12/pBH7 (SS-*sspO*-SUC2) | This study |
|  |  |  |
| ***E. festucae*** | | |
| PN2278 (Fl1) | Wild-type | Young et al. 2005 |
| PN3113 (Δ*gpiB* T111) | Fl1/∆*gpiB*::P*trpC*-*hph*; Hyg^R^ | This study |
| PN3114 (Δ*gpiB* T133) | Fl1/∆*gpiB*::P*trpC*-*hph*; Hyg^R^ | This study |
| PN3115 (Δ*gpiB* T148) | Fl1/∆*gpiB*::P*trpC*-*hph*; Hyg^R^ | This study |
| PN3116 (Δ*sspM* T52) | Fl1/∆*sspM*::P*trpC*-*hph*; Hyg^R^ | This study |
| PN3117 (Δ*sspM* T99) | Fl1/∆*sspM*::P*trpC*-*hph*; Hyg^R^ | This study |
| PN3118 (Δ*sspM* T163) | Fl1/∆*sspM*::P*trpC*-*hph*; Hyg^R^ | This study |
| PN3119 (Δ*sspN* T10) | Fl1/∆*sspN*::P*trpC*-*hph*; Hyg^R^ | This study |
| PN3120 (Δ*sspN* T30) | Fl1/∆*sspN*::P*trpC*-*hph*; Hyg^R^ | This study |
| PN3121 (Δ*sspN* T52) | Fl1/∆*sspN*::P*trpC*-*hph*; Hyg^R^ | This study |
| PN3122 (Δ*sspO* T78) | Fl1/∆*sspO*::P*trpC*-*hph*; Hyg^R^ | This study |
| PN3123 (Δ*sspO* T195) | Fl1/∆*sspO*::P*trpC*-*hph*; Hyg^R^ | This study |
| PN3124 (Δ*sspO* T210) | Fl1/∆*sspO*::P*trpC*-*hph*; Hyg^R^ | This study |
| *gpiB* OE T2 | Fl1/pBH29; Hyg^R^ | This study |
| *gpiB* OE T6 | Fl1/pBH29; Hyg^R^ | This study |
| *gpiB* OE T10 | Fl1/pBH29; Hyg^R^ | This study |
| *sspM* OE T1 | Fl1/pBH30; Hyg^R^ | This study |
| *sspM* OE T6 | Fl1/pBH30; Hyg^R^ | This study |
| *sspM* OE T10 | Fl1/pBH30; Hyg^R^ | This study |
| *sspN* OE T2 | Fl1/pBH31; Hyg^R^ | This study |
| *sspN* OE T4 | Fl1/pBH31; Hyg^R^ | This study |
| *sspN* OE T10 | Fl1/pBH31; Hyg^R^ | This study |
| *sspO* OE T2 | Fl1/pBH32; Hyg^R^ | This study |
| *sspO* OE T3 | Fl1/pBH32; Hyg^R^ | This study |
| *sspO* OE T7 | Fl1/pBH32; Hyg^R^ | This study |
| *sspM*-mCherry-NLS T4 | Fl1/pBH19, pBH28; Hyg^R^, Gen^R^ | This study |
| *sspM*-mCherry-NLS T5 | Fl1/pBH19, pBH28; Hyg^R^, Gen^R^ | This study |
| *sspM*-mCherry-NLS T10 | Fl1/pBH19, pBH28; Hyg^R^, Gen^R^ | This study |
| *sspN*-mCherry-NLS T2 | Fl1/pBH22, pBH28; Hyg^R^, Gen^R^ | This study |
| *sspN*-mCherry-NLS T5 | Fl1/pBH22, pBH28; Hyg^R^, Gen^R^ | This study |
| *sspN*-mCherry-NLS T8 | Fl1/pBH22, pBH28; Hyg^R^, Gen^R^ | This study |
| *sspO*-mCherry-NLS T2 | Fl1/pBH25, pBH28; Hyg^R^, Gen^R^ | This study |
| *sspO*-mCherry-NLS T6 | Fl1/pBH25, pBH28; Hyg^R^, Gen^R^ | This study |
| *sspO*-mCherry-NLS T9 | Fl1/pBH25, pBH28; Hyg^R^, Gen^R^ | This study |
| *gpiB-His T1* | Fl1/pBH56; Hyg^R^ | This study |
| *gpiB -His T2* | Fl1/pBH56; Hyg^R^ | This study |
| *sspM-His T1* | Fl1/pBH57; Hyg^R^ | This study |
| *sspM-His T2* | Fl1/pBH57; Hyg^R^ | This study |
| *sspN-His T1* | Fl1/pBH58; Hyg^R^ | This study |
| *sspN-His T2* | Fl1/pBH58; Hyg^R^ | This study |
| *sspO-His T1* | Fl1/pBH59; Hyg^R^ | This study |
| *sspO-His T2* | Fl1/pBH59; Hyg^R^ | This study |
|  |  |  |
| ***L. perenne*** | | |
| *L. perenne* cv. Samson | - | AgResearch |
|  |  |  |
| ***N. benthamiana*** | | |
| *N. benthamiana* |  | Mesarich Lab (Massey University) |
|  |  |  |
| ***N. tabacum*** | | |
| *N. tabacum* Wisconsin 38 |  | Mesarich Lab (Massey University) |
|  |  |  |
| **Plasmid** | **Characteristics** | **Reference** |
| PN1862 (pSF15.15) | pSP72 containing 1.4-kb *Hind*III P*trpC*-*hph* from pCB1004 cloned into *Sma*I site. Amp^R^; Hyg^R^; *Nco*I-free P*trpC*-*hph* | S. Foster |
| PN4241(pBV579) | pAN583 containing a 0.1 kb *Bsr*GI/*Bam*HI fragment containing NLS (three tandem repeats of the nuclear localization signal from simian virus large T-antigen) from pEBFP2-Nuc | Khang et al. 2010 |
| PN4111 (pPN94) | pSF14.14 containing 0.8-kb *Sa*lI/*Xba*I *tef* promoter in *Xho*I/*Xba*I site and 0.6-kb *EcoR*I/*Bgl*II T*trpC* in *Eco*RI/*Bgl*II site | Takemoto et al. 2006 |
| PN4276 (pSUC2TM13ORI) | Amp^R^, TRP+, -Met-*Eco*RI/*Xho*I-SUC2 | Jacobs et al., 1997 |
| PN4183 (pRS426) | *ori*(f1)-l*acZ*-T7 promoter-MCS (*Kpn*I-*Sac*I)-T3 promoter-*lacI*-*ori*(pMB1)-amp^R^-*ori* (2 micron), *URA3;* Amp^R^ | Christianson et al. 1992 |
| pICH86988 | Kan^R^, *lacZ* | Weber et al. 2011 |
| pBC302-3-R3a | pBC302-3 containing potato R3 gene; Kan^R^ | Melissa Guo |
| pBIN-Plus-Avr3a | pBIN-Plus containing *P. infestans* Avr3 gene; Kan^R^ | Melissa Guo |
| pBC302-3-INF1 | pBC302-3 containing *P. infestans* INF1 gene; Kan^R^ | Melissa Guo |
| pICH86966-N-3xFLAG-GFP | pICH86966 containing *gfp* with N-terminal 3xFLAG tag; Kan^R^ | Melissa Guo |
| pICH41021 | pUC19 containing plant PR1α-3xFLAG tag secretion signal; Kan^R^ | S. Marillonet |
| pBH1 | pRS426 containing 4.4 kb *Bg*lII/*Bam*HI *gpiB* deletion construct; Hyg^R^ | This study |
| pBH2 | pRS426 containing 4.1 kb B*gl*II/*Bgl*II *sspM* deletion construct; Hyg^R^ | This study |
| pBH3 | pRS426 containing 4.2 kb *Bgl*II/*Hind*III *sspN* deletion construct; Hyg^R^ | This study |
| pBH4 | pRS426 containing 4.1 kb *Bgl*II/*Bam*HI *sspO* deletion construct; Hyg^R^ | This study |
| pBH5 | pSUC2 containing the 57 bp *Eco*RI/*Xho*I *gpiB* secretion signal fragment | This study |
| pBH7 | pSUC2 containing the 54 bp *Eco*RI/*Xho*I *sspN* secretion signal fragment | This study |
| pBH9 | pSUC2 containing the 54 bp *Eco*RI/*Xho*I *sspO* secretion signal fragment | This study |
| pBH11 | pSUC2 containing the 54 bp *EcoR*I/*Xho*I *sspM* secretion signal fragment | This study |
| pBH12 | pPN94 containing 723 bp *B. cinerea* *tub* terminator insert amplified from pNR1 downstream of *hph*; Hyg^R^ | This study |
| pBH14 | pBH12 containing 3.4 kb P*tef*-*gpiB-mCherry* insert; Hyg^R^ |  |
| pBH18 | pBH12 containing 3.7 kb P*tef*-*sspM-mCherry* insert; Hyg^R^ |  |
| pBH19 | pBH12 containing 1.5 kb insert of P*sspM-sspM* fused to *mCherry-NLS* amplified from pBV597; Hyg^R^ | This study |
| pBH21 | pBH12 containing 3.5 kb P*tef*-*sspN-mCherry* insert; Hyg^R^ |  |
| pBH22 | pBH12 containing 1.4 kb insert of P*sspN*-*sspN* fused to *mCherry-NLS* amplified from pBV597; Hyg^R^ | This study |
| pBH24 | pBH12 containing 3.4 kb P*tef*-*sspO*-*mCherry* insert; Hyg^R^ |  |
| pBH25 | pBH12 containing 1.4 kb insert of P*sspO*-*sspO* fused to *mCherry-NLS* amplified from pBV597; Hyg^R^ | This study |
| pBH28 | pBH12 containing *nptII* replacing *hph* and *gfp;* Gen^R^ | This study |
| pBH29 | pBH12 containing 1.4 kb insert of P*tef-gpiB*; Hyg^R^ | This study |
| pBH30 | pBH12 containing 1.5 kb insert of *Ptef-sspM*; Hyg^R^ | This study |
| pBH31 | pBH12 containing 1.4 kb insert of *Ptef-sspN*; Hyg^R^ | This study |
| pBH32 | pBH12 containing 1.4 kb insert of *Ptef-sspO*; Hyg^R^ | This study |
| pBH34 | pBH12 containing x kb P*tef*-*gpiB* (N-terminus)-*mCherry*-*gpiB* (C-terminus) insert; Hyg^R^ |  |
| pBH44 | pICH86988 containing a 492 bp PRIα-*gpiB* insert, lacking *sspL* secretion signal | This study |
| pBH45 | pICH86988 containing a 638 bp PRIα-*sspM* insert, lacking *sspM* secretion signal | This study |
| pBH46 | pICH86988 containing a 498 bp PRIα-*sspN* insert, lacking *sspN* secretion signal | This study |
| pBH47 | pICH86988 containing a 429 bp PRIα-*sspO* insert, lacking *sspO* secretion signal | This study |
| pBH56 | pBH29 containing a 8xHis-tag 30 bp upstream of the *gpiB* omega site | This study |
| pBH57 | pBH30 containing a *sspM* C-terminal 8xHis-tag | This study |
| pBH58 | pBH31 containing a *sspN* C-terminal 8xHis-tag | This study |
| pBH59 | pBH32 containing a *sspO* C-terminal 8xHis-tag | This study |
|  |  |  |
|  |  |  |
|  |  |  |
|  |  |  |

**Table S2.** Primers used in this study.

| **Primers used for PCR.** | | |
| --- | --- | --- |
| **Name** | **Sequence (5’-3’)** | **Purpose** |
| BH1 | *GCCAGGGTTTTCCCAGTCACGACAGATCT*GACAAGATTGTCTCGCATCG | ∆*gpiB* 5’fragmen*t* |
| BH2 | GCTCCTTCAATATCAGTTCCAAGCTCAAGAGTGCGACAACAAAGC | ∆*gpiB* 5’fragmen*t* |
| BH3 | CGTCCGAGGGCAAAGGAATAGGTAGATCAAACAACACTGAACAGG | ∆*gpiB* 3’fragmen*t* |
| BH4 | ATAACAATTTCACACAGGAAACAGCGGATCCACGTACATGAAGACACGTTTG | ∆*gpiB* 3’fragmen*t* |
| BH5 | ACGCCAGGGTTTTCCCAGTCACGACAGATCTTCATGGTACGACGACTGCTC | ∆*sspO* 5’fragmen*t* |
| BH6 | GCTCCTTCAATATCAGTTCCAAGCTTTGGCGCCAGATATTTTCTC | ∆*sspO* 5’fragmen*t* |
| BH7 | CACTCGTCCGAGGGCAAAGGAATAGGCTTGAACTACGTGTAGGAC | ∆*sspO* 3’fragmen*t* |
| BH8 | ATAACAATTTCACACAGGAAACAGCGGATCCACAAACTTTCTGGCATTGGG | ∆*sspO* 3’fragmen*t* |
| BH9 | ACGCCAGGGTTTTCCCAGTCACGACAGATCTGCAGTCATTAGATGACATCGTG | ∆*sspM* 3’fragmen*t* |
| BH10 | CACTCGTCCGAGGGCAAAGGAATAGCGCTTGAGACTAAATGGGATG | ∆*sspM* 3’fragmen*t* |
| BH11 | GCTCCTTCAATATCAGTTCCAAGCTCTCCAACTGAGACCTACACTTT | ∆*sspM* 5’fragmen*t* |
| BH12 | ATAACAATTTCACACAGGAAACAGCGGATCCCTTTCGATCGGGTCTTGATG | ∆*sspM* 5’fragmen*t* |
| BH13 | ACGCCAGGGTTTTCCCAGTCACGACAGATCTCTCTCTTTGTCGCTCTTTGC | ∆*sspN* 3’fragmen*t* |
| BH14 | CACTCGTCCGAGGGCAAAGGAATAGATGGCATATCATATCCCAGCTC | ∆*sspN* 3’fragmen*t* |
| BH15 | GCTCCTTCAATATCAGTTCCAAGCTAGTTTATTCCGTCACACCCG | ∆*sspN* 5’fragmen*t* |
| BH16 | GATAACAATTTCACACAGGAAACAGCAAGCTTGCCTTTCAAGAGATACGTACG | ∆*sspN* 5’fragmen*t* |
| BH21 | GAATTCATGCGGTTCTTAAACGTCTTC | *sspN* secretion signal |
| BH22 | CTCGAGTGGGGCGGCTAATGCCAAGG | *sspN* secretion signal |
| BH25 | CATGCCTGCAGGTCGAGATC | sequencing of pSUC2T7M13ORI |
| BH26 | GTCCAATGCTAGTAGAGAAG | sequencing of pSUC2T7M13ORI |
| BH27 | GTCCAATGCTAGTAGAGAAG | sequencing of pSUC2T7M13ORI |
| BH28 | GGAAGAAAGATTTGACGACT | sequencing of pSUC2T7M13ORI |
| BH29 | CAACTCCACTCAATTCAGAG | sequencing of pSUC2T7M13ORI |
| BH30 | GTTTACGCTGTTAACACCAC | sequencing of pSUC2T7M13ORI |
| BH31 | TGCCTTACACTACTAGGATG | sequencing of pSUC2T7M13ORI |
| BH32 | TTACCAATGCTTAATCAGTG | sequencing of pSUC2T7M13ORI |
| BH33 | AAACTCTCAAGGATCTTACC | sequencing of pSUC2T7M13ORI |
| BH34 | ATGTCTGTTATTAATTTCAC | sequencing of pSUC2T7M13ORI |
| BH35 | TCAAAAATGCTAAGAAATAG | sequencing of pSUC2T7M13ORI |
| BH36 | TACAGAACAGAAATGCAACG | sequencing of pSUC2T7M13ORI |
| BH37 | CAAAAGCGCTCTGAAGTTCC | sequencing of pSUC2T7M13ORI |
| BH38 | GCTATCAAGTATAAATAGAC | sequencing of pSUC2T7M13ORI |
| BH39 | GAATCCCGATGTATGGGTTTG | sequencing of pSUC2T7M13ORI |
| BH40 | CTTGAAGTCCTGGAAGCTAG | sequencing of pSUC2T7M13ORI |
| BH41 | GTCGCTCTTATTGACCACAC | sequencing of pSUC2T7M13ORI |
| BH42 | CACTGGGGCCAGATGGTAAG | sequencing of pSUC2T7M13ORI |
| BH43 | TCCTCACCAAAACTGACTGC | ∆*gpiB* verification |
| BH44 | GAGAAGACTCGTGGTGATTTG | ∆*gpiB* verification |
| BH45 | GAACCGTCACAAGAGGAACC | ∆*sspM* verification |
| BH46 | GACATGCAACAAGACGATGG | ∆*sspM* verification |
| BH47 | GGATTGGTCCAAATTCATGG | ∆*sspN* verification |
| BH48 | CTGACAGCCTTTCAAGAGATAC | ∆*sspN* verification |
| BH49 | GAGAATGACGAGGGAGTGATG | ∆*sspO* verification |
| BH50 | CAGTGTCAGAGGAGCCAGAG | ∆*sspO* verification |
| BH59 | TATCCACGCCCTCCTACATC | deletion verification |
| BH60 | GTTGACGGCAATTTCGATG | deletion verification |
| BH61 | GAGAGCGCTATTTTACCAAC | sequencing of pSUC2T7M13ORI |
| BH62 | GGTAGTCTGAAGAAGCATCG | sequencing of pSUC2T7M13ORI |
| BH63 | GGAGTTGACTAATGTTGTGGG | sequencing of pSUC2T7M13ORI |
| BH64 | GACTGAGAATTCATGCAATTGACCACTCTCATTC | *gpiB* secretion signal |
| BH65 | GACGTACTCGAGGGTGTCGGCGAGAGCCATAGC | *gpiB* secretion signal |
| BH68 | GACTGAGAATTCATGAAGCTCACCACTACCCTC | *sspO* secretion signal |
| BH69 | GACGTACTCGAGGGGCGTTGCCACGACCAGAGTC | *sspO* secretion signal |
| BH70 | GACTGAGAATTCATGAAGGTCGTCTCCATCATCGCTGCTGCCCTG | *sspM* secretion signal |
| BH71 | GACGTACTCGAGGATTGCTGCGATGGTTGCGGAACTGCTGGCCAGGGCA | *sspM* secretion signal |
| BH72 | CTTCCGCTTCCTCGCTCACTG | pBH12/pPN94 amplification |
| BH75 | CAGTGAGCGAGGAAGCGGAAGGCTTGCTTAGCTTGATATCTG | pBH12 amplification |
| BH76 | CTGAAATCATCAAACAGCTTG | pBH12 amplification |
| BH78 |  |  |
| BH79 | GGAGGTGGAGGTTCTGGTGGAGGTGGATCTATGGTGAGCAAGGGCGAGGA | *mCherry* with linker |
| BH80 | CAAGCTGTTTGATGATTTCAGGCTTTAAGATCCTACCTTTCTC | *mCherry* |
| BH86 | CATAGATCCACCTCCACCAGAACCTCCACCTCCAGCGTGGAAACCCGCGGTGCAG | *sspM* for pBH19 |
| BH88 | CACATACGATTTAGGTGACACCTTGTCAGGAGCGCAAAGGTC | *sspM* for pBH19 |
| BH89 | CATAGATCCACCTCCACCAGAACCTCCACCTCCAAGGTACGATTGGTGACGATC | *sspN* for pBH22 |
| BH91 | CACATACGATTTAGGTGACACTAGCAAAGCCATTGAGCTTC | *sspN* for pBH22 |
| BH93 | CATAGATCCACCTCCACCAGAACCTCCACCTCCAGCCTTGCACTTGCCCGTG | *sspO* for pBH25 |
| BH94 | CACATACGATTTAGGTGACACGTGGTGTTTCTCTGTGCACTG | *sspO* for pBH25 |
| BH120 | CAAGCTGTTTGATGATTTCAGCTACATCAGAACAACAGCAAGC | *gpiB* for pBH29 |
| BH121 | CAAGCTGTTTGATGATTTCAGTCAAGCGTGGAAACCCGCGGTG | *sspM* for pBH30 |
| BH122 | CAAGCTGTTTGATGATTTCAGTTAAAGGTACGATTGGTGACGATC | *sspN* for pBH31 |
| BH123 | CAAGCTGTTTGATGATTTCAGTCAAGCCTTGCACTTGCCCGTGAA | *sspO* for pBH32 |
| BH165 | GGTCTCGCAAGGACACCAGCTCTGCCA | *gpiB* without secretion signal for pBH44 |
| BH166 | GGTCTCTAAGCTACATCAGAACAACAGCAAGC | *gpiB* without secretion signal for pBH44 |
| BH167 | GGTCTCGCAAGGCAATCGTCCCAGTCCAGCC | *sspM* without secretion signal for pBH45 |
| BH168 | GGTCTCTAAGCTAAGCGTGGAAACCCGCGGT | *sspM* without secretion signal for pBH45 |
| BH170 | GGTCTCGCAAGGCCCCAACAAAATCAGCTG | *sspN* without secretion signal for pBH46 |
| BH171 | GGTCTCTAAGCTAAAGGTACGATTGGTGACG | *sspN* without secretion signal for pBH46 |
| BH172 | GGTCTCGCAAGACGCCCACGCCAGACGAAG | *sspO* without secretion signal for pBH47 |
| BH173 | GGTCTCTAAGCTAAGCCTTGCACTTGCCCGT | *sspO* without secretion signal for pBH47 |
| BH176 | CATCACCATCACCATCACCATCACTAACTGAAATCATCAAACAGCTTG | pBH12 amplification, addition of His-tag |
| BH177 | TTAGTGATGGTGATGGTGATGGTGATGAGCGTGGAAACCCGCGGTG | *sspM* with His-tag |
| BH178 | TTAGTGATGGTGATGGTGATGGTGATGAAGGTACGATTGGTGACGATC | *sspN* with His-tag |
| BH179 | TTAGTGATGGTGATGGTGATGGTGATGAGCCTTGCACTTGCCCGTGAAG | *sspO* with His-tag |
| BH180 | GGGGTGATGGTGATGGTGATGGTGATGCCCCGGAGCAGTGGTGAC | *gpiB* with His-tag |
| BH181 | CATCACCATCACCATCACCATCACCCCGAACCAACCATCAAG | *gpiB* with His-tag |
| pRS426_F | GCTGTTTCCTGTGTGAAATTG | *pRS426* backbone |
| pRS426_R | GGGTTTTCCCAGTCACGAC | *pRS426* backbone |
| hph_F | AGCTTGGAACTGATATTGAAGG | *hph* for deletion constructs |
| hph_R | CGTCCGAGGGCAAAGGAATAG | *hph* for deletion constructs |
| **Primers used for RT-qPCR.** | | |
| BH128 | GTCTTCACTTCTCCTGCCGT | *gpiB* copy number/expression |
| BH129 | CTTGGTGGCATCGAGAGCA | *gpiB* copy number/expression |
| BH132 | GCAAATGCGGGTCCAACAAG | *sspM* copy number/expression |
| BH133 | GTCGTTGGGAGCATAACCGG | *sspM* copy number/expression |
| BH136 | TAGCCGCCCCAACAAAATGT | *sspN* copy number |
| BH137 | CAACGAGGGCATCAGTAGCA | *sspN* copy number |
| BH138 | GTCTTCATTCTTCTCCCAGCCT | *sspN* expression |
| BH139 | CCGTCAGGCTCAACGAGG | *sspN* expression |
| BH142 | GAAGCTCACCACTACCCTCG | *sspO* copy number/expression |
| BH143 | CCAGGATGTTGTCTCTCTGGC | *sspO* copy number/expression |
|  |  |  |

|  |  |
| --- | --- |
