# Supplementary material for: Analysis of Epichloë festucae small secreted proteins in the interaction with Lolium perenne
Source: PLoS One. 2019 Feb 13;14(2):e0209463. doi: 10.1371/journal.pone.0209463 (PMC6374014; doi:10.1371/journal.pone.0209463)
Supplement: S3 Table — (DOCX) [file pone.0209463.s009.docx]

**Table S3.** Primers used in this study.

| **Primers used for PCR.** | | |
| --- | --- | --- |
| **Name** | **Sequence (5’-3’)** | **Purpose** |
| BH1 | *GCCAGGGTTTTCCCAGTCACGACAGATCT*GACAAGATTGTCTCGCATCG | ∆*gpiB* 5’fragmen*t* |
| BH2 | GCTCCTTCAATATCAGTTCCAAGCTCAAGAGTGCGACAACAAAGC | ∆*gpiB* 5’fragmen*t* |
| BH3 | CGTCCGAGGGCAAAGGAATAGGTAGATCAAACAACACTGAACAGG | ∆*gpiB* 3’fragmen*t* |
| BH4 | ATAACAATTTCACACAGGAAACAGCGGATCCACGTACATGAAGACACGTTTG | ∆*gpiB* 3’fragmen*t* |
| BH5 | ACGCCAGGGTTTTCCCAGTCACGACAGATCTTCATGGTACGACGACTGCTC | ∆*sspO* 5’fragmen*t* |
| BH6 | GCTCCTTCAATATCAGTTCCAAGCTTTGGCGCCAGATATTTTCTC | ∆*sspO* 5’fragmen*t* |
| BH7 | CACTCGTCCGAGGGCAAAGGAATAGGCTTGAACTACGTGTAGGAC | ∆*sspO* 3’fragmen*t* |
| BH8 | ATAACAATTTCACACAGGAAACAGCGGATCCACAAACTTTCTGGCATTGGG | ∆*sspO* 3’fragmen*t* |
| BH9 | ACGCCAGGGTTTTCCCAGTCACGACAGATCTGCAGTCATTAGATGACATCGTG | ∆*sspM* 3’fragmen*t* |
| BH10 | CACTCGTCCGAGGGCAAAGGAATAGCGCTTGAGACTAAATGGGATG | ∆*sspM* 3’fragmen*t* |
| BH11 | GCTCCTTCAATATCAGTTCCAAGCTCTCCAACTGAGACCTACACTTT | ∆*sspM* 5’fragmen*t* |
| BH12 | ATAACAATTTCACACAGGAAACAGCGGATCCCTTTCGATCGGGTCTTGATG | ∆*sspM* 5’fragmen*t* |
| BH13 | ACGCCAGGGTTTTCCCAGTCACGACAGATCTCTCTCTTTGTCGCTCTTTGC | ∆*sspN* 3’fragmen*t* |
| BH14 | CACTCGTCCGAGGGCAAAGGAATAGATGGCATATCATATCCCAGCTC | ∆*sspN* 3’fragmen*t* |
| BH15 | GCTCCTTCAATATCAGTTCCAAGCTAGTTTATTCCGTCACACCCG | ∆*sspN* 5’fragmen*t* |
| BH16 | GATAACAATTTCACACAGGAAACAGCAAGCTTGCCTTTCAAGAGATACGTACG | ∆*sspN* 5’fragmen*t* |
| BH21 | GAATTCATGCGGTTCTTAAACGTCTTC | *sspN* secretion signal |
| BH22 | CTCGAGTGGGGCGGCTAATGCCAAGG | *sspN* secretion signal |
| BH25 | CATGCCTGCAGGTCGAGATC | sequencing of pSUC2T7M13ORI |
| BH26 | GTCCAATGCTAGTAGAGAAG | sequencing of pSUC2T7M13ORI |
| BH27 | GTCCAATGCTAGTAGAGAAG | sequencing of pSUC2T7M13ORI |
| BH28 | GGAAGAAAGATTTGACGACT | sequencing of pSUC2T7M13ORI |
| BH29 | CAACTCCACTCAATTCAGAG | sequencing of pSUC2T7M13ORI |
| BH30 | GTTTACGCTGTTAACACCAC | sequencing of pSUC2T7M13ORI |
| BH31 | TGCCTTACACTACTAGGATG | sequencing of pSUC2T7M13ORI |
| BH32 | TTACCAATGCTTAATCAGTG | sequencing of pSUC2T7M13ORI |
| BH33 | AAACTCTCAAGGATCTTACC | sequencing of pSUC2T7M13ORI |
| BH34 | ATGTCTGTTATTAATTTCAC | sequencing of pSUC2T7M13ORI |
| BH35 | TCAAAAATGCTAAGAAATAG | sequencing of pSUC2T7M13ORI |
| BH36 | TACAGAACAGAAATGCAACG | sequencing of pSUC2T7M13ORI |
| BH37 | CAAAAGCGCTCTGAAGTTCC | sequencing of pSUC2T7M13ORI |
| BH38 | GCTATCAAGTATAAATAGAC | sequencing of pSUC2T7M13ORI |
| BH39 | GAATCCCGATGTATGGGTTTG | sequencing of pSUC2T7M13ORI |
| BH40 | CTTGAAGTCCTGGAAGCTAG | sequencing of pSUC2T7M13ORI |
| BH41 | GTCGCTCTTATTGACCACAC | sequencing of pSUC2T7M13ORI |
| BH42 | CACTGGGGCCAGATGGTAAG | sequencing of pSUC2T7M13ORI |
| BH43 | TCCTCACCAAAACTGACTGC | ∆*gpiB* verification |
| BH44 | GAGAAGACTCGTGGTGATTTG | ∆*gpiB* verification |
| BH45 | GAACCGTCACAAGAGGAACC | ∆*sspM* verification |
| BH46 | GACATGCAACAAGACGATGG | ∆*sspM* verification |
| BH47 | GGATTGGTCCAAATTCATGG | ∆*sspN* verification |
| BH48 | CTGACAGCCTTTCAAGAGATAC | ∆*sspN* verification |
| BH49 | GAGAATGACGAGGGAGTGATG | ∆*sspO* verification |
| BH50 | CAGTGTCAGAGGAGCCAGAG | ∆*sspO* verification |
| BH59 | TATCCACGCCCTCCTACATC | deletion verification |
| BH60 | GTTGACGGCAATTTCGATG | deletion verification |
| BH61 | GAGAGCGCTATTTTACCAAC | sequencing of pSUC2T7M13ORI |
| BH62 | GGTAGTCTGAAGAAGCATCG | sequencing of pSUC2T7M13ORI |
| BH63 | GGAGTTGACTAATGTTGTGGG | sequencing of pSUC2T7M13ORI |
| BH64 | GACTGAGAATTCATGCAATTGACCACTCTCATTC | *gpiB* secretion signal |
| BH65 | GACGTACTCGAGGGTGTCGGCGAGAGCCATAGC | *gpiB* secretion signal |
| BH68 | GACTGAGAATTCATGAAGCTCACCACTACCCTC | *sspO* secretion signal |
| BH69 | GACGTACTCGAGGGGCGTTGCCACGACCAGAGTC | *sspO* secretion signal |
| BH70 | GACTGAGAATTCATGAAGGTCGTCTCCATCATCGCTGCTGCCCTG | *sspM* secretion signal |
| BH71 | GACGTACTCGAGGATTGCTGCGATGGTTGCGGAACTGCTGGCCAGGGCA | *sspM* secretion signal |
| BH72 | CTTCCGCTTCCTCGCTCACTG | pBH12/pPN94 amplification |
| BH75 | CAGTGAGCGAGGAAGCGGAAGGCTTGCTTAGCTTGATATCTG | pBH12 amplification |
| BH76 | CTGAAATCATCAAACAGCTTG | pBH12 amplification |
| BH78 |  |  |
| BH79 | GGAGGTGGAGGTTCTGGTGGAGGTGGATCTATGGTGAGCAAGGGCGAGGA | *mCherry* with linker |
| BH80 | CAAGCTGTTTGATGATTTCAGGCTTTAAGATCCTACCTTTCTC | *mCherry* |
| BH86 | CATAGATCCACCTCCACCAGAACCTCCACCTCCAGCGTGGAAACCCGCGGTGCAG | *sspM* for pBH19 |
| BH88 | CACATACGATTTAGGTGACACCTTGTCAGGAGCGCAAAGGTC | *sspM* for pBH19 |
| BH89 | CATAGATCCACCTCCACCAGAACCTCCACCTCCAAGGTACGATTGGTGACGATC | *sspN* for pBH22 |
| BH91 | CACATACGATTTAGGTGACACTAGCAAAGCCATTGAGCTTC | *sspN* for pBH22 |
| BH93 | CATAGATCCACCTCCACCAGAACCTCCACCTCCAGCCTTGCACTTGCCCGTG | *sspO* for pBH25 |
| BH94 | CACATACGATTTAGGTGACACGTGGTGTTTCTCTGTGCACTG | *sspO* for pBH25 |
| BH120 | CAAGCTGTTTGATGATTTCAGCTACATCAGAACAACAGCAAGC | *gpiB* for pBH29 |
| BH121 | CAAGCTGTTTGATGATTTCAGTCAAGCGTGGAAACCCGCGGTG | *sspM* for pBH30 |
| BH122 | CAAGCTGTTTGATGATTTCAGTTAAAGGTACGATTGGTGACGATC | *sspN* for pBH31 |
| BH123 | CAAGCTGTTTGATGATTTCAGTCAAGCCTTGCACTTGCCCGTGAA | *sspO* for pBH32 |
| BH165 | GGTCTCGCAAGGACACCAGCTCTGCCA | *gpiB* without secretion signal for pBH44 |
| BH166 | GGTCTCTAAGCTACATCAGAACAACAGCAAGC | *gpiB* without secretion signal for pBH44 |
| BH167 | GGTCTCGCAAGGCAATCGTCCCAGTCCAGCC | *sspM* without secretion signal for pBH45 |
| BH168 | GGTCTCTAAGCTAAGCGTGGAAACCCGCGGT | *sspM* without secretion signal for pBH45 |
| BH170 | GGTCTCGCAAGGCCCCAACAAAATCAGCTG | *sspN* without secretion signal for pBH46 |
| BH171 | GGTCTCTAAGCTAAAGGTACGATTGGTGACG | *sspN* without secretion signal for pBH46 |
| BH172 | GGTCTCGCAAGACGCCCACGCCAGACGAAG | *sspO* without secretion signal for pBH47 |
| BH173 | GGTCTCTAAGCTAAGCCTTGCACTTGCCCGT | *sspO* without secretion signal for pBH47 |
| BH176 | CATCACCATCACCATCACCATCACTAACTGAAATCATCAAACAGCTTG | pBH12 amplification, addition of His-tag |
| BH177 | TTAGTGATGGTGATGGTGATGGTGATGAGCGTGGAAACCCGCGGTG | *sspM* with His-tag |
| BH178 | TTAGTGATGGTGATGGTGATGGTGATGAAGGTACGATTGGTGACGATC | *sspN* with His-tag |
| BH179 | TTAGTGATGGTGATGGTGATGGTGATGAGCCTTGCACTTGCCCGTGAAG | *sspO* with His-tag |
| BH180 | GGGGTGATGGTGATGGTGATGGTGATGCCCCGGAGCAGTGGTGAC | *gpiB* with His-tag |
| BH181 | CATCACCATCACCATCACCATCACCCCGAACCAACCATCAAG | *gpiB* with His-tag |
| pRS426_F | GCTGTTTCCTGTGTGAAATTG | *pRS426* backbone |
| pRS426_R | GGGTTTTCCCAGTCACGAC | *pRS426* backbone |
| hph_F | AGCTTGGAACTGATATTGAAGG | *hph* for deletion constructs |
| hph_R | CGTCCGAGGGCAAAGGAATAG | *hph* for deletion constructs |
| **Primers used for RT-qPCR.** | | |
| BH128 | GTCTTCACTTCTCCTGCCGT | *gpiB* copy number/expression |
| BH129 | CTTGGTGGCATCGAGAGCA | *gpiB* copy number/expression |
| BH132 | GCAAATGCGGGTCCAACAAG | *sspM* copy number/expression |
| BH133 | GTCGTTGGGAGCATAACCGG | *sspM* copy number/expression |
| BH136 | TAGCCGCCCCAACAAAATGT | *sspN* copy number |
| BH137 | CAACGAGGGCATCAGTAGCA | *sspN* copy number |
| BH138 | GTCTTCATTCTTCTCCCAGCCT | *sspN* expression |
| BH139 | CCGTCAGGCTCAACGAGG | *sspN* expression |
| BH142 | GAAGCTCACCACTACCCTCG | *sspO* copy number/expression |
| BH143 | CCAGGATGTTGTCTCTCTGGC | *sspO* copy number/expression |
|  |  |  |

|  |  |
| --- | --- |
