# Supplementary material for: Analysis of Epichloë festucae small secreted proteins in the interaction with Lolium perenne
Source: PLoS One. 2019 Feb 13;14(2):e0209463. doi: 10.1371/journal.pone.0209463 (PMC6374014; doi:10.1371/journal.pone.0209463)
Supplement: S7 Table — (DOCX) [file pone.0209463.s013.docx]

**S6 Table. Vertical transmission of *ssp* mutants in *L. perenne*.**

| **Strain** | **No of inoculated plants** | **No of infected plants** | **No. of vernalized plants** | **No. of plants with inflorescences** | **No of inflorescences per plant** | **Infected ovaries** | **No. of seeds immunoblotted** | **No. of infected seeds** |
| --- | --- | --- | --- | --- | --- | --- | --- | --- |
| Δ*gpiB* T111 | 20 | 8 | 5 | 1 | 13 | not infected | 30 | 0 |
| Δ*gpiB* T133 | 20 | 11 | 5 | 1 | 2 | infected | 6 | 2 |
| Δ*gpiB* T148 | 20 | 7 | 5 | 2 | 8/9 | not infected | 7 | 0 |
| Δ*sspM* T52 | 20 | 7 | 5 | 3 | 3/6/13 | Infected | 30 | 14 |
| Δ*sspM* T99 | 20 | 8 | 5 | 1 | 4 | infected | 30 | 11 |
| Δ*sspM* T163 | 20 | 5 | 5 | 2 | 11/4 | Infected | 30 | 0 |
| Δ*sspN* T10 | 20 | 6 | 5 | 1 | 11 | not infected | 30 | 6 |
| Δ*sspN* T30 | 20 | 5 | 5 | 3 | 3/1/9 | infected | 30 | 5 |
| Δ*sspN* T52 | 20 | 10 | 5 | 2 | 9/2 | infected | 10 | 3 |
| Δ*sspO* T78 | 20 | 11 | 5 | 1 | 7 | not infected | 30 | 0 |
| Δ*sspO* T195 | 20 | 11 | 5 | 1 | 5 | not infected | 30 | 0 |
| Δ*sspO* T210 | 20 | 11 | 5 | 0 | - | - | - | - |
